# Contrasting Photosensitized Processes of Ru(II) Polypyridyl Structural Isomers Containing Linear and Hooked Intercalating Ligands Bound to Guanine-Rich DNA

**DOI:** 10.1021/acs.jpcb.4c04129

**Published:** 2024-08-06

**Authors:** Mark Stitch, Rosie Sanders, Igor V. Sazanovich, Michael Towrie, Stanley W. Botchway, Susan J. Quinn

**Affiliations:** † School of Chemistry, University College Dublin, Dublin 4 D04 V1W8, Ireland; ‡ Central Laser Facility, Research Complex at Harwell, STFC Rutherford Appleton Laboratory, Harwell Science and Innovation Campus, Didcot, Oxfordshire OX11 0QX, U.K.

## Abstract

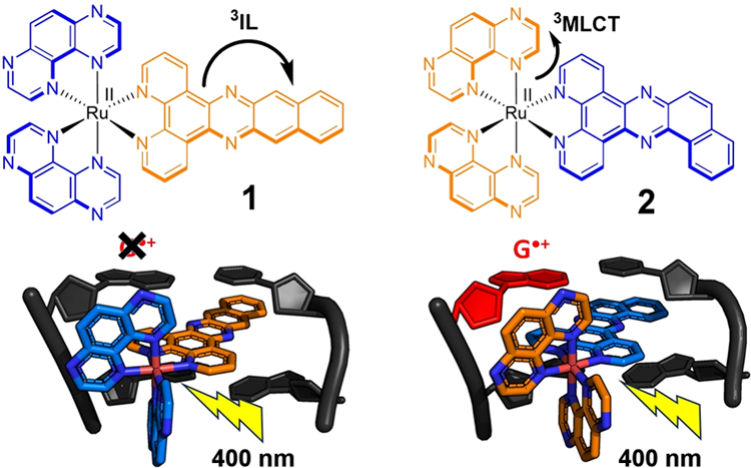

The DNA binding and cellular uptake of the lambda enantiomer of two bis-tetraazaphenanthrene (TAP) Ru(II) polypyridyl complexes containing either a linear dppn (**1**) or a hooked bdppz (**2**) benzodipyridophenazine ligand are reported, and the role of different charge-transfer states of the structural isomers in the photo-oxidation of guanine is explored. Both complexes possess characteristic metal-to-ligand charge-transfer (MLCT) bands between 400 and 500 nm and emission at ca. 630 nm in an aerated aqueous solution. Transient visible absorption (TrA) spectroscopy reveals that 400 nm excitation of **1** yields a dppn-based metal-to-ligand charge-transfer (MLCT) state, which in turn populates a dppn intraligand (^3^IL) state. In contrast, photoexcitation of **2** results in an MLCT state on the TAP ligand and not the intercalating bdppz ligand. Both **1** and **2** bind strongly to double-stranded guanine-rich DNA with a loss of emission. Combined TrA and time-resolved infrared (TRIR) spectroscopy confirms formation of the guanine radical cation when **2** is bound to the d(G_5_C_5_)_2_ duplex, which is not the case when **1** is bound to the same duplex and indicates a different mechanism of action in DNA. Utilizing the long-lived triplet excited lifetime, we show good uptake and localization of **2** in live cells as well as isolated chromosomes. The observed shortening of the excited-state lifetime of **2** when internalized in cell chromosomes is consistent with DNA binding and luminescent quenching due to guanine photo-oxidation.

## 1 Introduction

Ruthenium polypyridyl complexes show great potential as therapeutic photosensitizers due to their affinity for DNA and tunable photophysical properties.^1^ Typically, the mechanism of photodamage is either via formation of reactive oxygen species (ROS) through electron transfer in a type I photodynamic therapy (PDT) process or more commonly by an energy-transfer mechanism that sensitizes singlet oxygen (^1^O_2_) formation in a type II process.^2^ An excellent example of the latter is the Ru(II) polypyridyl complex (TLD1433), which has entered phase II clinical trial for treating high-risk nonmuscle invasive bladder cancer.^3,4^ The activity of TLD1433 arises due to the extended conjugation on the α-terthienyl group, which provides access to a long-lived triplet intraligand (^3^IL) charge-transfer excited state, which acts as a highly effective sensitizer of ^1^O_2_.^3,4^ Related to this, DNA photocleavage has also been observed for [Ru(bpy)_2_dppn]Cl_2_ through the low-lying and long-lived intraligand (IL) ^3^ππ* state localized on the extended dppn (benzo[*i*]dipyrido[3,2-a:2′,3′-*c*]phenazine) ligand.^5−8^ The formation of this excited state has also been seen for related Ru-dppn complexes^9−12^ as well as for the dppn-containing rhodium complexes.^13^ However, recently, the phenanthroline-containing bis-dppn complex ([Ru(dppn)_2_phen](PF_6_)_2_) has been shown to disrupt mitochondrial respiration.^14^ In the case of these dppn complexes, the activity toward DNA is attributed to the formation of singlet oxygen. In contrast, excitation of the MLCT band in ruthenium polypyridyl complexes containing strong π-accepting 1,4,5,8-tetraazaphenanthrene (TAP) ligands has been shown to lead to DNA damage through the mechanism of direct oxidation of guanine by the Ru complex.^15^ This effect has been enhanced for complexes containing the extended dipyrido[3,2-a:2′,3′-*c*]phenazine (dppz) ligand, e.g. [Ru(TAP)_2_dppz]Cl_2_, which binds strongly to a variety of double-stranded DNA sequences.^16−18^ We are interested in exploring the use of this direct electron-transfer mechanism as an additional approach for the development of photodynamic therapy agents. To this end, time-resolved infrared (TRIR) spectroscopy has proven to be an excellent technique to both resolve the excited-state dynamics of the metal complex and provide information regarding the site of DNA damage. It is particularly powerful for the study of DNA photodamage as it allows detection of the guanine radical cation in double-stranded DNA (ca. 1700 cm^–1^),^19^ as well as providing information about the nucleobases in the binding site, which are identified by diagnostic bleach bands formed due to perturbation caused by the formation of the metal complex excited state.^20^ We have extensively used this “site effect” to report on the site of photo-oxidation in a variety of DNA systems in solution and in crystals.^17,18,20,21^

**Figure 1:**
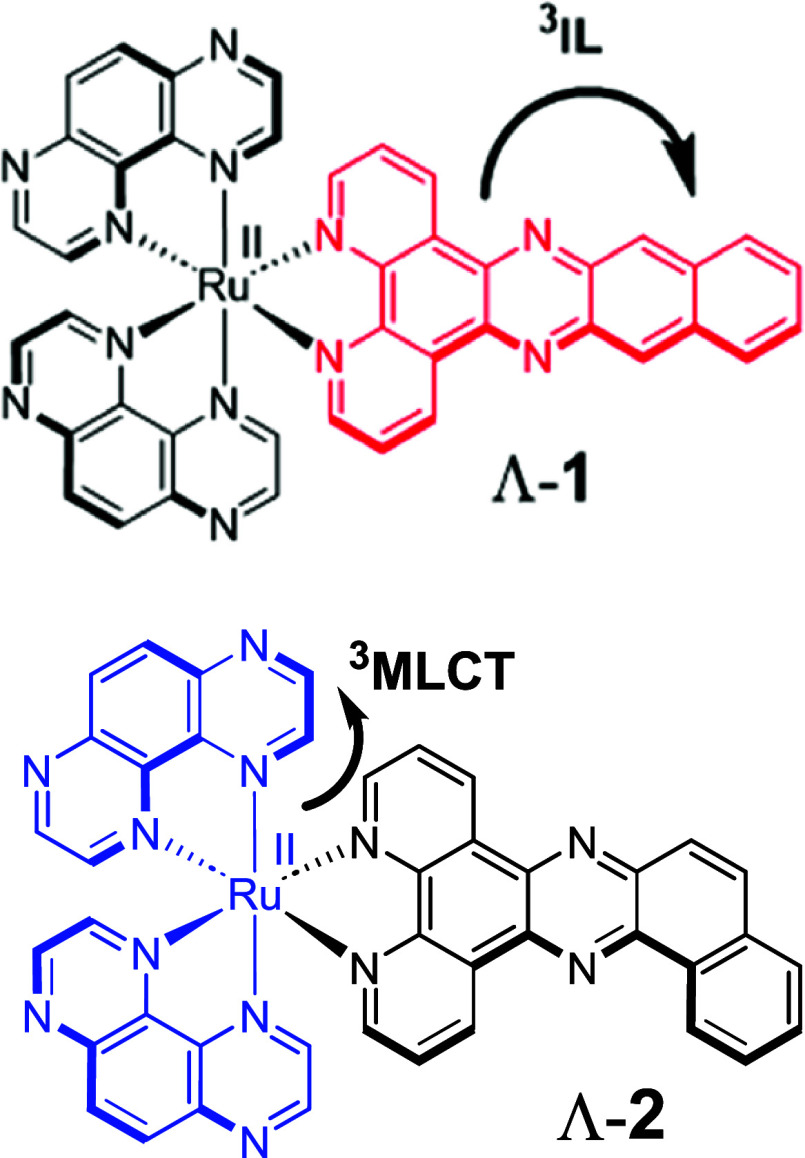
Structures of complexes **1** and **2** indicate the transition associated with the lowest excited-state emission.

DNA binding can be enhanced by modifying the intercalating ligand through ring substitution or extension.^22^ For example, complexes containing the dppn ligand exhibit strong intercalative binding interactions with duplex DNA (binding constants ≥10^6^ M^–1^) via π-stacking with adjacent base pairs.^5,11^ Related to this, high DNA affinity has also been observed for complexes containing the “hooked” pdppz ligand ([2,3-h]dipyrido[3,2-a:2′,3′-*c*]phenazine).^23^ Notably, these extended aromatic complexes are more readily internalized by live cells (without the need for a transporting agent) due to their increased lipophilicity.^8,10,24^ We previously studied the formation of the guanine radical cation arising from the strong binding of the lambda enantiomer of [Ru(TAP)_2_dppz]^2+^ to duplexes formed from the self-complementary 5′-dG_5_C_5_–3′ sequence.^25^ Developing from this, the current study investigates the lambda enantiomers of two new TAP complexes containing two structural isomers of an extended polypyridyl ligand in the [Ru(TAP)_2_LL]^2+^ system, where LL is either a linear dppn or hooked bdppz (bdppz = benzo[*h*]dipyrido[3,2-a:2′,3′-*c*]phenazine) ligand, to yield complexes [Ru(TAP)_2_dppn]^2+^ (**1**) and [Ru(TAP)_2_(bdppz)] (**2**), see Figure 1. These systems were chosen to allow comparison of their DNA binding interactions and their ability to photo-oxidize DNA through direct one electron transfer. Additionally, we were interested to study the role of the ^3^IL and ^3^MLCT excited states where the excited state may be located either on the intercalating ligand or on the groove-bound TAP ligand.

## 2 Methods

### 2.1 Synthesis

The TAP ligand was prepared using a known literature procedure.^26^ The dppn and bdppz ligands were synthesized from 1,10-phenanthroline-5,6-dione using literature procedures.^6^ The purity of all ligands was confirmed by ^1^H NMR. All other chemicals employed were reagent-grade quality from commercial sources and were used without further purification.

#### 2.2.1. Synthesis of [Ru(TAP)_2_(dppn)][PF_6_]_2_ (**1**)

Ru(TAP)_2_Cl_2_ (300 mg, 0.56 mmol, 1 equiv) was suspended with dppn (215 mg, 0.56 mmol, 1 equiv) in an ethylene glycol mixture (4 mL) in a microwave vial. The mixture was irradiated under an inert atmosphere for 45 min at 473 K and then filtered to remove any unreacted Ru(TAP)_2_Cl_2_. The reaction solution was then cooled to room temperature before the addition of a saturated aqueous solution of NH_4_PF_6_ (2 mL). The suspension was collected by filtration and washed with deionized water and diethyl ether. Purification was achieved by column chromatography (Alumina, 40:4:1 MeCN/H_2_O/sat. aq. NaNO_3_), which afforded the nitrate salt. The PF_6_ salt of the complex was reformed. This was converted to the water-soluble chloride salt by swirling the PF_6_ complex in MeOH (20 mL) in the presence of Amberlite ion-exchange resin (Cl form) for 1 h. The suspension was filtered, and the solvent removed under reduced pressure. The crude product was further purified using a CM-Sephadex C25 column and a salt gradient between 0.01 and 0.1 M to isolate the pure product [Ru(TAP)_2_(dppn)]·2Cl (96 mg, 31% yield). ^**1**^**H NMR (500 MHz, DMSO-*****d***_**6**_**)** δ 9.71–9.62 (m, 2H), 9.28 (s, 2H), 9.13 (d, *J* = 2.9 Hz, 2H), 9.08 (d, *J* = 2.8 Hz, 2H), 8.68 (s, 4H), 8.53 (d, *J* = 2.8 Hz, 2H), 8.47 (dd, *J* = 6.3, 3.1 Hz, 4H), 8.37–8.31 (m, 2H), 7.95 (dd, *J* = 8.2, 5.4 Hz, 2H), 7.80 (dd, *J* = 6.8, 3.2 Hz, 2H). **HRMS-ESI**^**+**^ (*m*/*z*): calc. for [RuC_42_H_24_N_12_Cl]^+^: expected: 833.0981 found: 833.0983 M^+^[Cl^–^].

#### 2.2.2. Synthesis of [Ru(TAP)_2_(bdppz)][Cl]_2_ (**2**)

Ru(TAP)_2_Cl_2_ (200 mg, 0.37 mmol, 1 equiv) was suspended with bdppz (143 mg, 0.37 mmol, 1 equiv) in an ethylene glycol mixture (4 mL) in a microwave vial. The mixture was irradiated under an inert atmosphere for 45 min at 473 K and then filtered to remove any unreacted Ru(TAP)_2_Cl_2_. The reaction solution was then cooled to room temperature, and the complex was purified and isolated as described above to yield the pure product [Ru(TAP)_2_(bdppz)]·2Cl (19 mg, 6% yield). ^**1**^**H NMR (500 MHz, deuterium oxide)** δ 9.92 (d, *J* = 8.2 Hz, 1H), 9.69 (d, *J* = 7.9 Hz, 1H), 9.44 (s, 1H), 8.91 (d, *J* = 2.8 Hz, 4H), 8.57 (s, 4H), 8.40 (d, *J* = 2.9 Hz, 2H), 8.30 (d, *J* = 2.9 Hz, 2H), 8.25 (d, *J* = 9.1 Hz, 1H), 8.13 (s, 4H), 8.0 (d, 1H), 7.92–7.78 (m, 3H). **HRMS-ESI**^**+**^ (*m*/*z*): calc. for [RuC_42_H_24_N_12_]^+^: expected: 798.1289 found: 798.1296 M^+^.

### 2.2 Materials and Instruments

The oligonucleotides were synthesized, desalted, and purified (by gel filtration) by Eurogentec (Liege, Belgium). Salmon testes’ natural DNA was purchased from Sigma-Aldrich. Guanine monophosphate GMP (ε_260 nm_ = 11,800 M^–1^ cm^–1^, nucleobase), d(G_5_C_5_)_2_ (ε_260 nm_ = 190,300 M^–1^ cm^–1^, double-stranded), and salmon testes DNA (st-DNA) (ε_260 nm_ = 6600 M^–1^ cm^–1^, nucleobase) concentrations were determined spectrophotometrically.

### 2.3 Measurements

^1^H NMR spectra were obtained on a Varian VnmrS 400 MHz spectrometer. All electrospray ionization mass spectrometry (ESI-MS) studies were performed using an Agilent 6546 Q-TOF series LC/MS system. UV/vis absorption spectra were recorded on a Varian Cary 200 or a Varian Cary 50 spectrophotometer. Steady-state luminescence spectra were recorded on a Varian Cary Eclipse. Circular dichroism measurements were recorded on a JASCO J-810 spectropolarimeter. Infrared spectra were recorded on a Nicolet Avatar spectrometer. Samples were loaded into a demountable Harrick cell (Harrick Scientific Products Inc., New York) assembled with 2 mm diameter CaF_2_ plates (Crystran, Ltd., U.K.), separated by a 50 μm Teflon spacer in D_2_O, and recorded on a N_2_-flushed transmission accessory. Each spectrum is an average of 32 scans.

#### 2.3.1. DNA Titrations

The concentration of DNA was determined using the molar absorbance at 260 nm for st-DNA (6600 M^–1^ cm^–1^/nucleotide) and 5′-GCG-CGC-GCG-CGC-3′ (190,300 M^–1^ cm^–1^/double strand). UV/vis and emission titrations were carried out at [**1**] and [**2**] 1.5 (±0.5) × 10^–5^ M at 298 K by monitoring changes in the absorption and emission spectra of the complexes upon successive additions of aliquots of DNA, in potassium phosphate buffer (50 mM, pH 7). The results are quoted using the concentration of DNA expressed as a concentration of nucleobase [DNA] to Ru ratio ([DNA]: Ru ratio).

#### 2.3.2. DNA Binding Constant Calculations

The intrinsic binding method described by Bard et al.^27^ in the equation shown below (eq 1) was used to fit the emission data. The binding constant of the [**1**]^2+^ and [**2**]^2+^ enantiomers binding to DNA structures was determined by fitting eq 2 to a nonlinear plot of (*I*_a_ – *I*_f_)/(*I*_b_ – *I*_f_) vs [DNA], for the molar emission (with [DNA] concentration in nucleotides, fitted using Origin 2023 software).
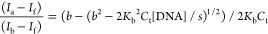
1
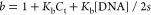
2where *K* is the equilibrium binding constant in M^–1^, *C*_t_ is the total metal complex concentration (changes to this are only due to dilution by the addition of DNA), *s* is the binding site size, and [DNA] is the concentration of DNA in nucleotides. *I*_f_ is the molar emission of the free metal complex (*I*_f_ = initial *E*_m_/*C*_t_), *I*_a_ is the apparent molar emission (*I*_a_ = apparent *E*_m_/*C*_t_), and *I*_b_ is the molar emission of the metal complex in the fully bound form (*I*_b_ = final *E*_m_/*C*_t_), and *E*_m_ is the emission.

#### 2.3.3. Time-Resolved Spectroscopy

TRIR spectroscopy measurements were conducted on the ULTRA and LIFETIME apparatus at the Central Laser Facility (STFC Rutherford Appleton Laboratory, Harwell, U.K.).^28^ During the experiments, the samples were raster-scanned in the *x* and *y* directions to minimize photodamage and re-excitation effects. The samples were excited at 400 nm for the Ru complexes. For the acquisition of the spectra, the polarization of the pump pulses at the sample was at the “magic” angle relative to the probe and was attenuated to between 400 nJ and 1 μJ.

#### 2.3.4. Cell Culture and Lifetime Imaging

All cell culture reagents, unless otherwise stated, were purchased from Thermo Fisher Scientific (Gibco). Both Chinese Hamster Ovary (CHO) and Henrietta Lacks (HeLa) cancer cell lines were from cryo preservation within the Research Complex at Harwell. The initial stock was purchased from ECACC (U.K.). Cells were grown in phenol red free DMEM/F-12 (Dulbecco′s modified Eagle Medium/Nutrient Mixture F-12) supplemented with 10% fetal calf serum (FCS), 5 mM glutamine/1% glutamax (Gibco, Life Technologies, U.K.), and 1% penicillin–streptomycin. Cells were maintained at a density of 1 × 10^5^ cells in a T-25 flask and passaged every 48–60 h, grown under humidified 5% CO_2_ in air at 37 °C, and regularly tested for mycoplasma contamination. For probe labeling experiments, cells in the exponential growth phase were seeded at a density of 5 × 10^4^ cells/mL in 8-well or 32 mm glass bottom chamber slides. The compounds were added to 70–90% confluent cells to a final concentration of 50–65 μM and were imaged from 3 to 24 h of the compounds being added. For organelle co-staining, LysoTracker Blue (Thermo Fisher, DND-22), ER Tracker green (Thermo Fisher) and NucBlue (Thermo Fisher) were used according to the manufacturers instructions.

Confocal images were taken using a Nikon EC2 scanning unit attached to an inverted Nikon Ti-E microscope. The confocal scanning unit was equipped with a 405 nm laser with a variable repetition rate, 70 ps pulse width (Becker and Hickl, Germany). Phosphorescence lifetime imaging microscopy (PLIM) images were acquired with a 60× (NA 1.20) water or 100× (NA 1.49) oil immersion objective. PLIM data were acquired following 405 nm (40 ps diode laser, variable repetition rate of 1–80 MHz variable) excitation. The laser and scanhead are coupled to a time-correlated single photon counting (TCSPC) SPC150 module equipped with an HPM100-42 detector (spectral sensitivity of 25% at 640–800 nm) running the TCSPC software v 9.77. A 485 nm together with 600 nm long-pass or a bandpass (3 mm BG39, 330–620 nm) filter was used to eliminate the excitation wavelength as well as to selectively observe organelle staining (below 600 nm) or compound (**2**) only (above 600 nm). The PLIM technique used here is the beam-blanking method. In this mode, the laser is allowed to run at 80 MHz during the pixel dwell time of the scanning system, followed by fast switching off during the frame clock flyback. This provided a very good phosphorescence excitation and emission detection than simply using a low repetition rate (kHz) pulse to match that of the lifetime expected. Cells were labeled with 50–65 μM **2** prior to imaging and incubated at 37 °C for 3 or 24 h.

## 3 Results and Discussion

### 3.1 Synthesis and Spectroscopic Characterization of **1** and **2**

The structural isomers [Ru(TAP)_2_dppn][Cl_2_] (**1**) and [Ru(TAP)_2_(bdppz)][Cl_2_] (**2**) were prepared in one-step reaction steps, by adapting previously reported literature methods (Scheme S1). Briefly, a stoichiometric amount of bdppz or dppn was reacted with [Ru(TAP)_2_(Cl)_2_]. Counterion metathesis with NH_4_PF_6_ yielded the hexafluorophosphate salts, which were subsequently exchanged with chloride ions by treatment over Amberlite to yield **1**^**2+**^ and **2**^**2+**^ as orange solids, whose identities were confirmed by NMR spectroscopy and mass spectrometry (Figures S1 and S2). The enantiomers were resolved by chiral chromatography, eluting with a (−)-*O*,*O*′-dibenzoyl-l-tartrate mobile phase, and circular dichroism was used to confirm the chiral separation of the Λ enantiomer (Figure S3).

**Figure 2:**
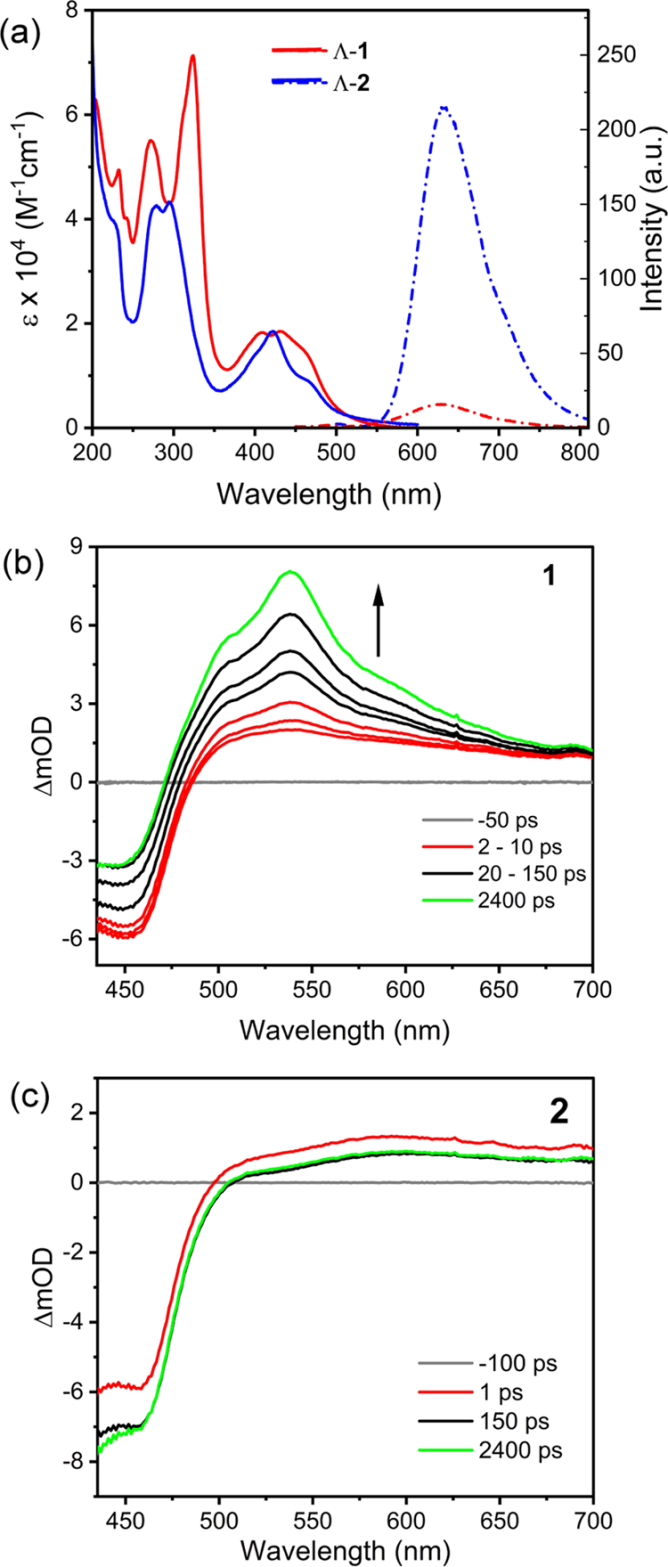
(a) UV–visible absorption and emission spectra of chloride salts **1** (λ_ex_ = 420 nm) and **2** (λ_ex_ = 420 nm) in aqueous solution. ps-TrA of (b) **1** showing the grow-in of the triplet state and (c) **2** recorded after 400 nm excitation in aqueous solution (400 nJ, 150 fs).

The UV–vis absorption spectra of **1** and **2** recorded in aqueous solution show polypyridyl ligand-centered (^1^LC) transitions between 250 and 300 nm, see Figure 2a. For **1**, additional ^1^LC transitions associated with the dppn ligand are observed at 323 nm and characteristic metal-to-ligand charge-transfer (^1^MLCT) bands are observed between 400 and 500 nm.^5,15^ Whereas, the spectrum of **2** shows Ru(II) dπ → π* ^1^MLCT bands at 422 nm due to bdppz and at 460 nm due to TAP, which is more π-accepting in nature, see Figure 2a. Complex **1** is found to be weakly emissive at 628 nm. This is attributed to the ability of the dppn ligand to facilitate nonradiative decay from a lower energy ^3^IL state.^5^ In contrast, **2** is found to show strong phosphorescence at 630 nm in aerated aqueous solution (Figure 2a), which is characteristic of the TAP-based ^3^MLCT emission also observed for the parent [Ru(TAP)_2_dppz]Cl_2_ complex.^29^

The time-resolved transient visible absorption (TrA) spectra of **1** and **2** recorded upon 400 nm excitation in D_2_O are shown in Figure 2b,c. The TrA spectrum of **1** recorded at 2 ps shows a bleach band at 450 nm indicating the loss of the MLCT ground state (GS) and a broad transient band between 500 and 650 nm associated with the ^3^MLCT excited state, which is expected to form by rapid intersystem crossing from the ^1^MLCT excited state. By 2 ns a new intense band at 540 nm is observed to grow in, which is attributed to the population of the intraligand ^3^ππ* state by internal conversion from the ^3^MLCT state, see Figure 2b.^6^ The appearance of this band (540 nm) has previously been reported for the free dppn ligand in CH_3_CN and for the [Ru(bpy)_2_dppn](PF_6_)_2_ complex.^5,6^ Analysis of the kinetics reveals that the population of the ^3^ππ* occurs with a rate constant of ca. 1.5 × 10^10^ s^–1^ (Figure S4). This process is observed to be enhanced in organic solvent with a slightly greater rate constant determined as 2.0 × 10^10^ s^–1^ (Figure S4). The IL process can also be observed from the TRIR spectra, which show the decay of the strong transient at 1457 cm^–1^ on the ns time scale^2^ and the grow-in of new transient bands at 1437 and 1481 cm^–1^ (Figure S5). Time-resolved measurements reveal contrasting behavior for **2**. The TrA measurement indicates that the dynamics of **2** are dominated by a TAP-based ^3^MLCT state, which absorbs with a characteristic broad positive transient feature at ca. 600 nm with no evidence of an intraligand transition observed (Figure 2c). This is also reflected in the TRIR spectra, which show formation of the TAP-based ^3^MLCT species (1457 cm^–1^).^30^ The presence of an appreciable 1457 cm^–1^ signal at >100 ns (Figure S5b) is consistent with emission lifetime of 820 ns recorded in aerated aqueous solution for the TAP-based ^3^MLCT species in the dppz complex.^30^ In summary, the TrA confirms that photoexcitation of **1** results in an MLCT state localized on the intercalating dppn ligand; in contrast, visible excitation of **2** yields an MLCT state localized on the groove-bound TAP ligand. Notably, in the TRIR for **1** and **2**, there is an absence of transient or bleach bands of significant intensity in the characteristic DNA region above 1625 cm^–1^.

### 3.2 Visible Absorption and Emission Studies of DNA Interactions

**Figure 3:**
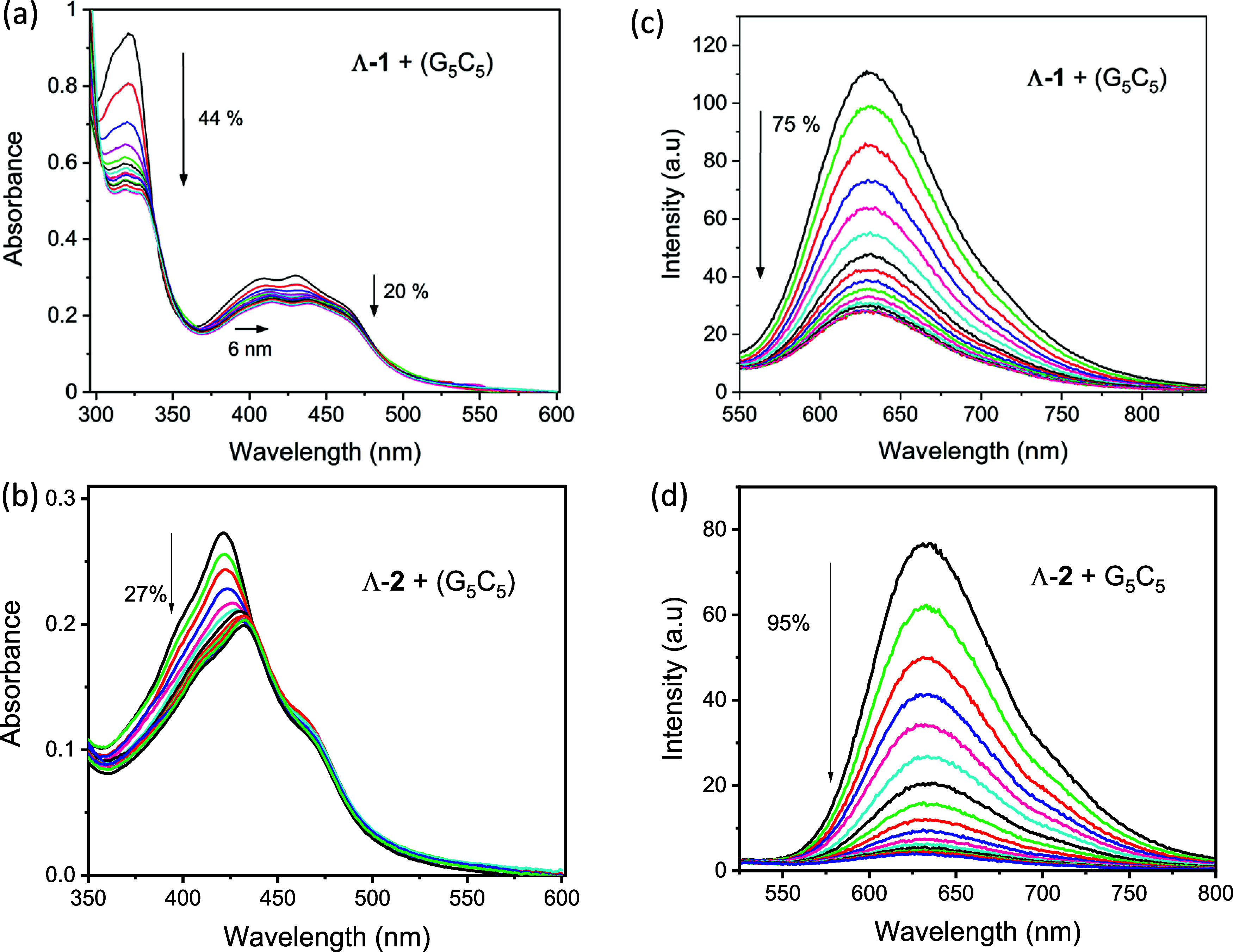
(a) UV–visible absorption and (c) luminescence spectra of Λ-**1** (16.5 μM) titrated against increasing concentrations of G_5_C_5_ (0–2 mM) in 50 mM phosphate buffer at pH 7. (b) Absorption and (d) luminescence spectra of Λ-**2** (15 μM) titrated against increasing concentrations of d(G_5_C_5_)_2_ (0–2 mM). Note the slit width of 20 nm for 3c and 5 nm for 3d.

The emission of **1** and **2** is quenched in the presence of increasing concentration of guanine monophosphate (Figure S6). Significant quenching is also observed in the presence of double-stranded DNA oligonucleotide formed by the self-complementary 5′-dGGGGGCCCCC-3′ (G_5_C_5_) sequence, which was chosen because of the greater susceptibility of sequential guanine bases to photo-oxidation, where 5′-GGG-3′ > 5′-GG-3′ > 5′-G-3′.^31,32^ The addition of G_5_C_5_ to Λ-**1** resulted in a large hypochromism (40–45%) in the ^1^LC band at 323 nm (Figure 3a), indicating strong π-stacking interactions between the intercalating dppn ligand and the surrounding nucleobases. This was accompanied by a significant decrease in the intensity of the ^1^MLCT of Λ-**1** (16–20%).

**Figure 4:**
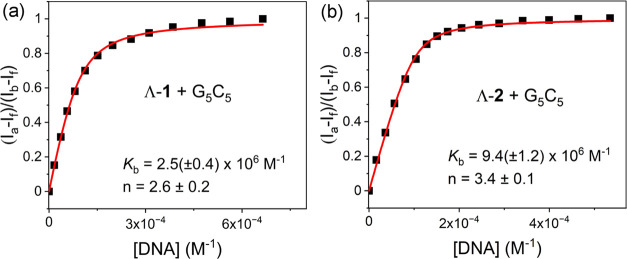
Determination of the binding constant for (a) Λ-**1** and (b) Λ-**2** in the presence of G_5_C_5_ DNA in 50 mM phosphate buffer. Plots of (*I*_a_ – *I*_f_)/(*I*_b_ – *I*_f_) fitting at ca. 600 nm vs [DNA] (per nucleobase) and nonlinear curve fitting of the data (**—**) using the method of Bard et al.^27^

In the case of Λ-**2**, the addition of G_5_C_5_ resulted in distinct changes in the intensity of the MLCT band which were accompanied by a red shift (10–12 nm) in the absorbance, see Figure 3b. Binding of the complexes to G_5_C_5_ DNA is accompanied by a 75% loss in emission at 628 nm for Λ-**1** (Figure 3c) and a 95% loss in emission at 630 nm for Λ-**2** (Figure 3d). Analysis of the emission data indicates that both complexes bind strongly to DNA but that Λ-**2** has a slightly greater affinity (*K*_b_ = 9.4 ± 1.2 × 10^5^ M^–1^) for G_5_C_5_ compared to Λ-**1** (*K*_b_ = 2.5 ± 0.6 × 10^6^ M^–1^), see Figure 4 and Table 1. Significantly less emission quenching is found when the complexes are added to natural salmon testes DNA (st-DNA), with 46% quenching observed for Λ-**1** and 58% quenching observed for Λ-**2**. This is attributed to binding of the complexes to AT regions in the natural DNA structure, which has 58% AT content, see Figure S7.

**Table 1:** Bard Binding Affinity Calculated for Changes in Emission at ca. 600 nm in terms of DNA Base Pair

DNA	binding constant	binding site size	*R*^2^
Λ-**1** + st-DNA (Em)	2.9(±0.2) × 10^7^ M^–1^	1.3(±0.1)	0.99
Λ-**1** + (G_5_C_5_) (Em)	2.5(±0.4) × 10^6^ M^–1^	2.6(±0.2)	0.99
Λ-**2** + st-DNA (Em)	1.1(±0.4) × 10^6^ M^–1^	1.8(±0.2)	0.99
Λ-**2** + (G_5_C_5_) (Em)	9.4(±1.2) × 10^6^ M^–1^	3.4(±0.1)	0.99

### 3.3 Visible Absorption and Infrared Time-Resolved Studies of DNA Interactions

**Figure 5:**
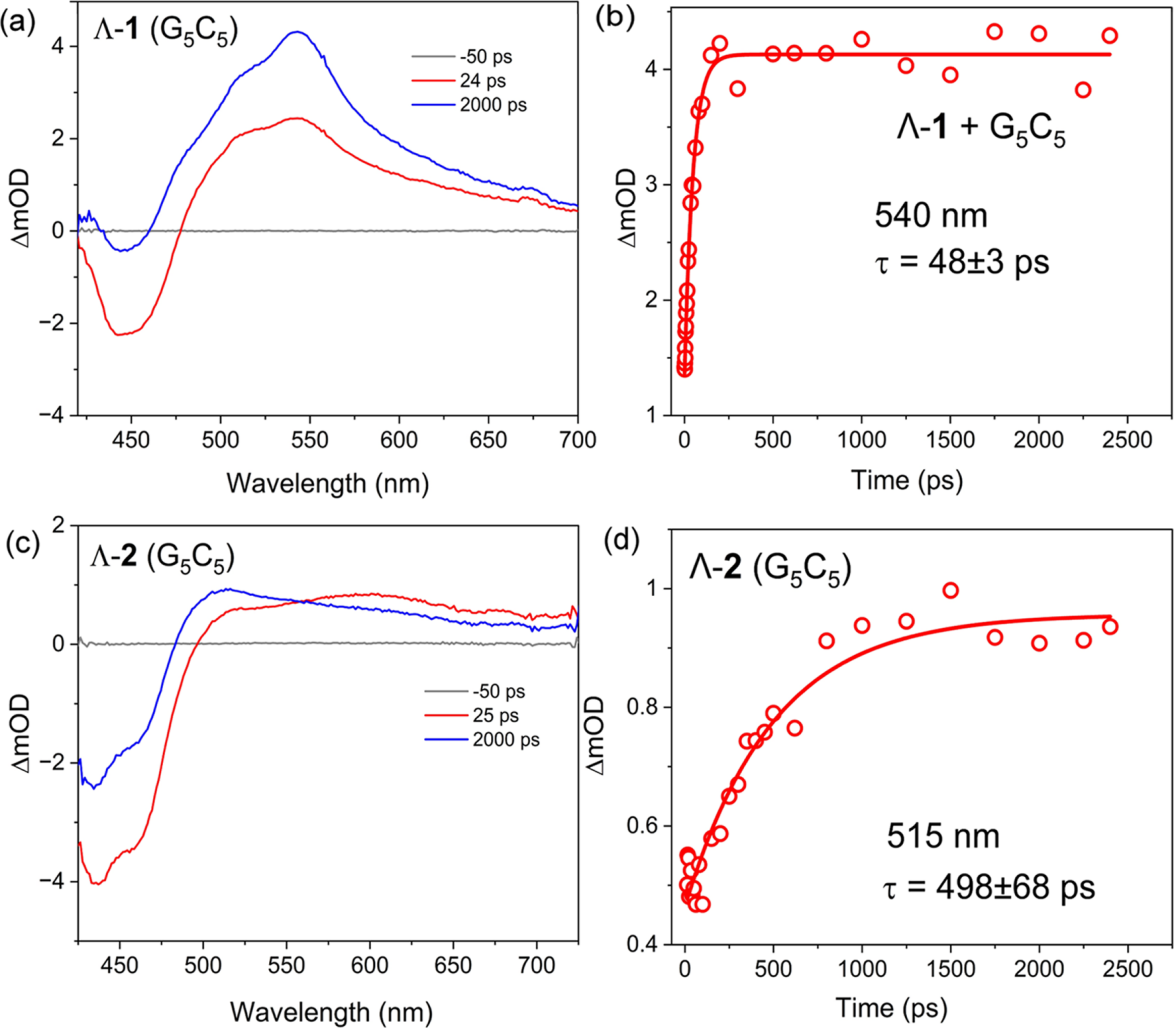
TrA and spectra of (a) 200 μM Λ-**1** and (c) 200 μM Λ-**2** in the presence of 300 μM d(G_5_C_5_)_2_ recorded at 24 or 25 and 2000 ps, 50 mM (λ_ex_= 400 nm, 400 nJ, 150 fs), all in 50 mM phosphate buffer (D_2_O). Kinetic fitting of the TrA transient at (b) 540 nm for Λ-**1** and (d) 515 nm for Λ-**2**.

The origin of emission quenching observed for Λ-**1** and Λ-**2** bound to G-rich duplex DNA was investigated using picosecond-to-second time-resolved absorption spectroscopy. The TrA spectra (1–2400 ps) recorded after 400 nm excitation of Λ-**1** bound to GC DNA are dominated by the “grow in” of the 540 nm band assigned to the ^3^ππ* dppn state (Figure 5a) and are observed to be very similar to the spectrum recorded in D_2_O (see Figure S8). Analysis of the kinetics reveals a rate constant for the formation of the ^3^ππ* similar to that observed in acetonitrile (2.0 ± 0.1 × 10^10^ s^–1^), see Figures 5b and S4. The similarity of the rate constant and spectrum indicates that the formation of the ^3^ππ* state persists, and is indeed facilitated, when the complex is bound to DNA, see Figure S9d. In contrast, the TrA spectrum of Λ-**2** in the presence of d(G_5_C_5_)_2_ shows a clear “grow in” of a transient band at 515 nm (τ = 498 ± 68 ps), see Figure 5c,d, which is not observed for the complex alone (Figure 2c). This 515 nm band has previously been observed for the formation of [Ru^II^(TAP)(TAP^•–^)(dppz)]^+^,^18,33^ and is therefore assigned to the formation of the reduced metal complex [Ru^II^(TAP)(TAP^•–^)(bdppz)]^+^. Notably, for Λ-**1**, the 515 nm feature is not readily observed but instead the TrA spectrum is observed to be similar to that observed in D_2_O (Figure S8).

**Figure 6:**
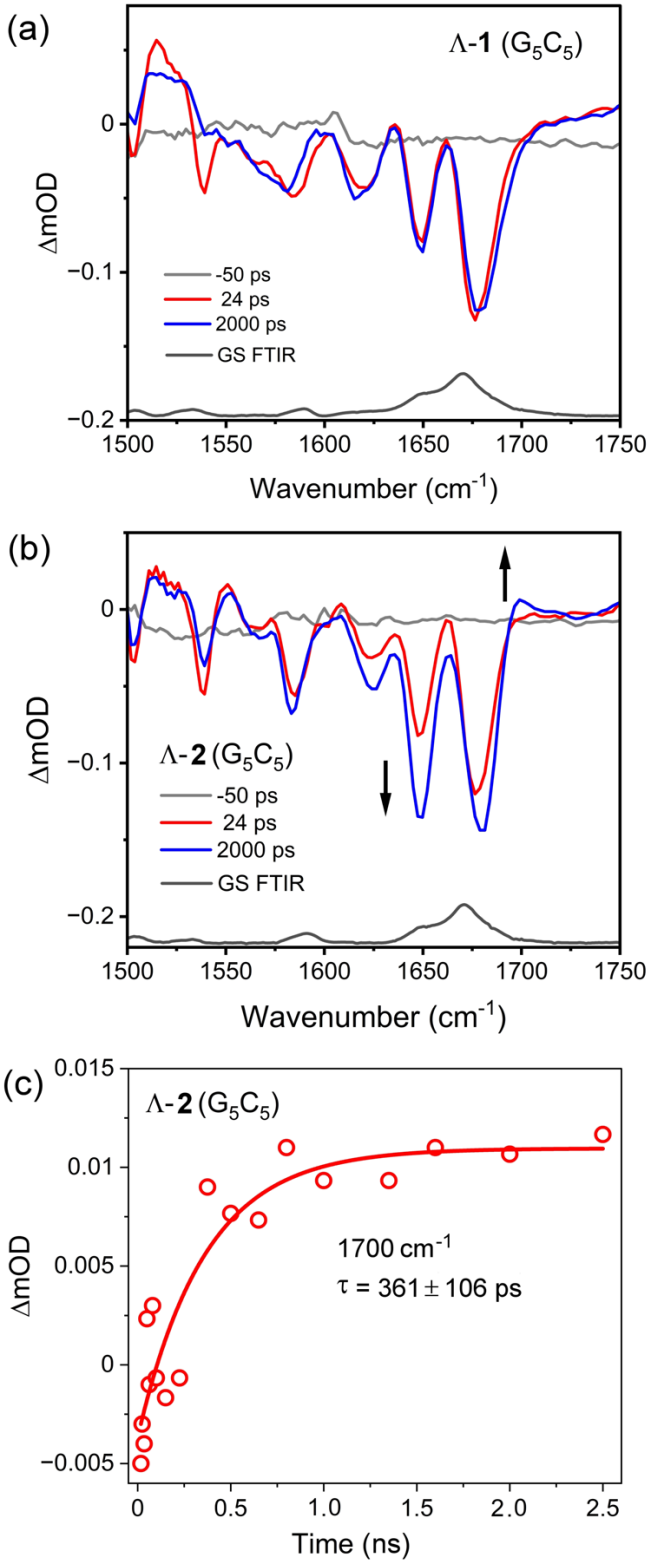
TRIR spectra of (a) 200 μM Λ-**1** and (b) 200 μM Λ-**2** recorded in the presence of 300 μM d(G_5_C_5_)_2_ at 24 and 2000 ps, 50 mM (λ_ex_= 400 nm, 1 μJ, 150 fs), all in 50 mM phosphate buffer (D_2_O). (c) Kinetic fitting of the TRIR transient at 1700 cm^–1^ for Λ-**2** in the presence of 300 μM d(G_5_C_5_)_2_.

The TRIR spectra of Λ-**1** in the presence of d(G_5_C_5_)_2_ recorded between 2 and 2000 ps show the evolution of the complex-based transient band at ca. 1460 cm^–1^ (see Figure 6a and Figure S10), which is similar to that observed for the complex alone in D_2_O (Figure S5a) and appears to track the ^3^IL process revealed in the TrA spectrum (Figure 5a). At 24 ps, clear bleach bands at 1650 and 1680 cm^–1^ are observed to be arising from the carbonyl vibrations of cytosine and guanine, respectively, see Figure 6a. The appearance of these bleach bands is attributed to the “site effect”, reflecting a perturbation of the nucleobases by the formation of the excited state of Λ-**1**.^2,3^ At early time <24 ps, some evolution of the nucleobase bleaches is observed (Figure S10a). However, it is notable that little to no evolution of the DNA bleach bands at 1600–1700 cm^–1^ is observed between 24 and 2000 ps, which is the time scale associated with the evolution of the ^3^ππ* state. This is interesting, as it suggests that the population of the ^3^MLCT excited state causes a more significant perturbation than the subsequent ^3^IL process. It is also noteworthy that no significant changes are observed in the spectrum in the region of ca. 1700 cm^–1^ where the formation of the characteristic (G^•+^) transient band is measured.

**Scheme 1:**
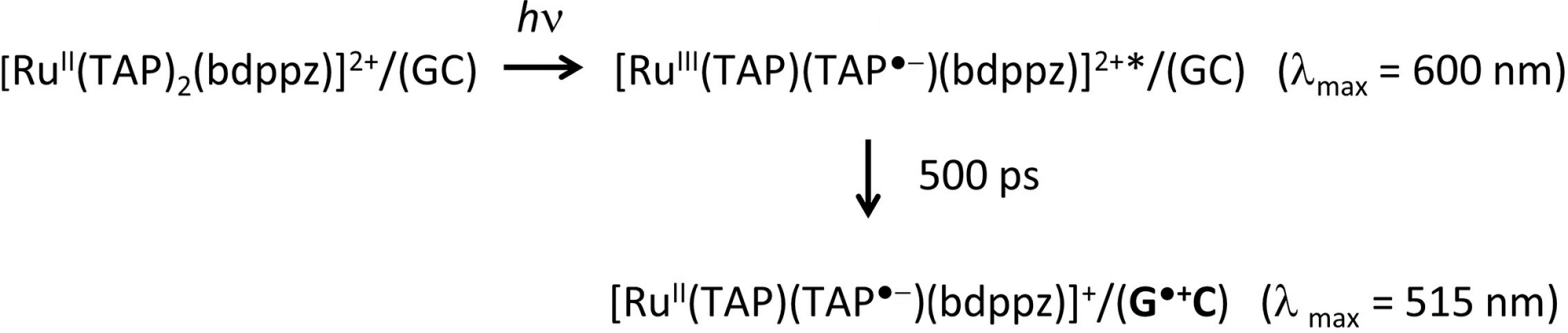
Photoreduction of Λ-**2** by an Electron Transfer from a Guanine Base (G) in d(G_5_C_5_)_2_

In contrast, the TRIR spectrum of Λ-**2** reveals a “grow in” of the characteristic bleach bands of C (1650 cm^–1^) and G (1680 cm^–1^) between 24 and 2000 ps (τ = 361 ± 106 ps), see Figure 6b. Critically, the appearance of a transient band at ca. 1700 cm^–1^ characteristic of the G^•+^ is observed to “grow in” with a τ of 361 ± 106 ps, which is on a comparable time scale as the grow-in of the 515 nm transient (τ = 498 ± 68 ps), see Figure 6c. The mechanism for this process is summarized in Scheme 1.

While the emission of both complexes is found to be reduced in the presence of guanine containing DNA, the time-resolved studies suggest that this is occurring by two different mechanisms. In the case of Λ-**2**, both TrA and TRIR confirm that quenching arises due to direct electron transfer. However, in the case is of Λ-**1**, the TRIR spectra do not provide evidence of electron transfer from guanine to DNA but instead suggest that binding to DNA either results in energy transfer or enhances the nonradiative decay process from the dppn ^3^ππ state.

### 3.4 Cellular Studies

**Figure 7:**
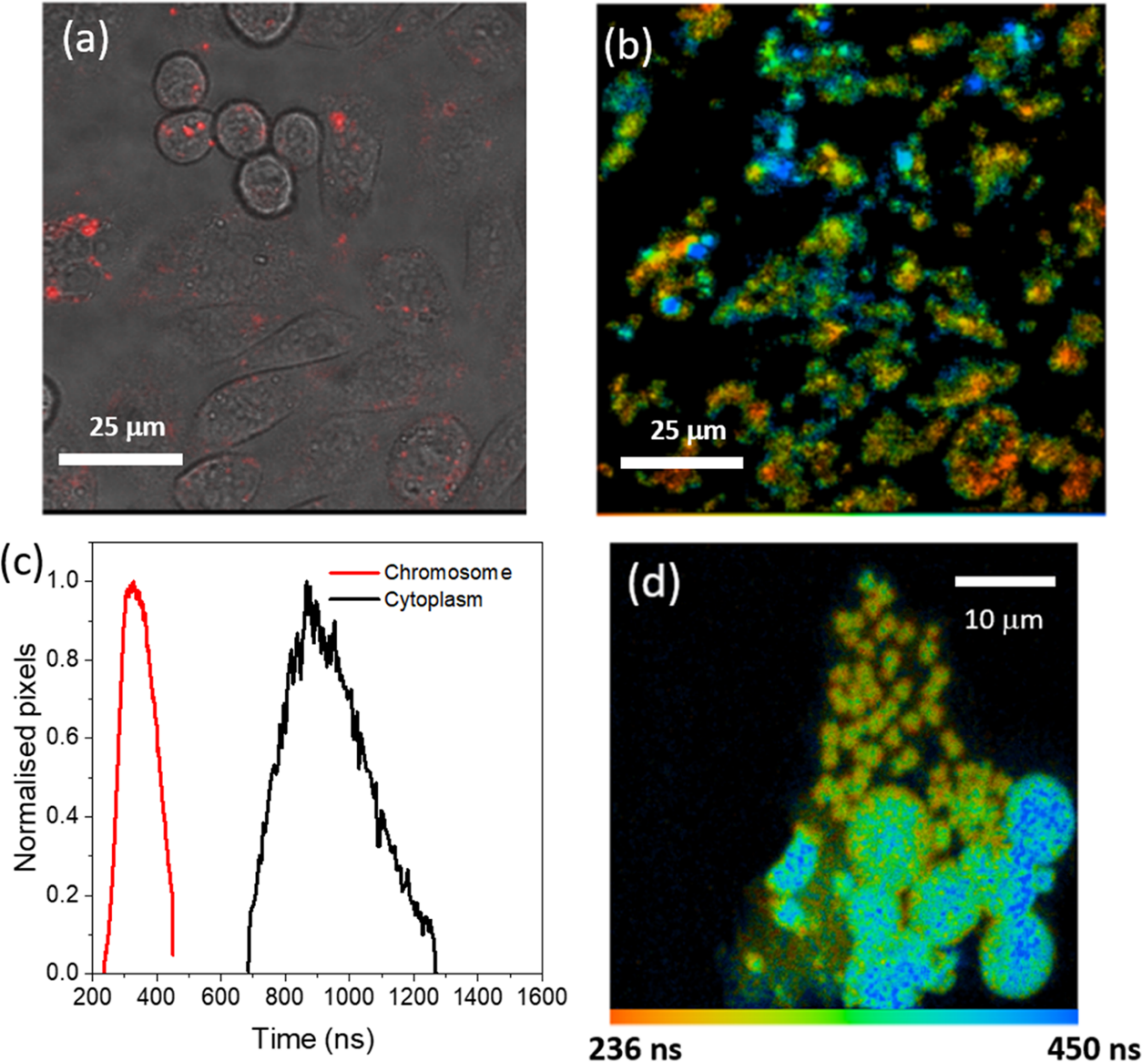
Confocal and PLIM imaging of live CHO cells and chromosomes incubated with complex **2** at room temperature. (a) Confocal image for CHO cells incubated for 24 h with 50 μM of **2** (60× microscope objective). (b) PLIM channel (λ_ex_ = 405 nm/λ_detection_ = 450 nm long pass) and (c) lifetime distribution curves of internalized **2**. (d) PLIM of isolated nuclei and chromosomes following excitation with 405 nm emission and the lifetime of phosphorescence at 650 nm. The same lifetime colour coding is used in (b) and (d).

Considering the potential of the complexes to bind DNA structures as well as future therapeutic applications, we investigated their uptake and internalization in live cells and the impact on their emission and excited-state properties. While both complexes **1** and **2** were observed to be internalized by CHO epithelial cells and HeLa, the intrinsic weaker emission of **1** proved challenging to study and so the imaging study focused on complex **2**. Laser scanning confocal microscopy following 405 nm excitation with emission beyond 650 nm after 3 and 24 h incubation revealed good cellular uptake and distribution of **2** throughout the cell (Figure S12). The steady-state confocal images clearly show localization in substructures of the cytoplasm with the majority of the probe emission detected being outside the nucleus as shown by a Hoechst co-staining (Figures 7a and S13). The more visible subnuclear localization is highly likely to be in the Golgi or endoplasmic reticulum (ER) and possibly lysosomes.

Phosphorescence lifetime imaging microscopy (PLIM) was used to study the cellular uptake of **2**. Notably, PLIM has the advantage of detecting emission on a longer time scale, which is ideally suited to measuring the triplet emissive states of transition metal complexes. We previously developed and demonstrated the use of a single instrument to achieve both FLIM and PLIM.^34^ When combined with confocal and multiphoton microscopy, FLIM–PLIM via time-correlated single photon counting (TCSPC) detection on a pixel-by-pixel basis provides very good spatial and temporal resolution. Thus, FLIM–PLIM–TCSPC achieves imaging over decades of time scales to provide highly useful data on the microenvironment that is not accessible in any other single method. The significant advantage of using lifetime imaging and, in particular, phosphorescence is that the long-lived nature of the probe can be used as further contrast and a clear indication of its presence within the cellular environment. The PLIM of **2** was measured after 3 and 24 h incubation with CHO and HeLa cells. At 3 h, the solution (cell culture media) outside the cells shows a higher lifetime (ca. 800 ns, blue) than when inside the cells (400–600 ns, green) highlighted by the blue ring around cells, Figure S12a. The significant differences in the distribution of the emission intensities in the confocal image (Figure 7a) and the PLIM image (Figure 7b) are attributed to significant quenching in the cell environment. While the significant quenching of **2** in cells leads to a low single photon count rate of the PLIM technique and a reduction in the overall image intensity, the long-lived lifetime provides a clear indication of the presence of the complex. There are several factors that could result in this luminescence lifetime reduction observed in the cytoplasm including (i) cellular protein interactions, (ii) pH quenching, and (iii) most significantly DNA binding. We also observed a further reduction of the emission lifetime of **2** to ca. 260 ns when the complex localized within the nucleus, see Figure S13b, which is likely a result of cellular DNA interactions.

To determine the level of quenching of the probe due to binding to nuclear DNA, we employed the use of mammalian cell (CHO) chromosomes and intact nuclei (Figures 7c and S14). We note that complex **2** is highly efficient in labeling both isolated nuclei and chromosomes, albeit with quenching of the emission. Furthermore, the lifetime (PLIM) of the chromosomes is lower (∼300 ns) compared to that of the isolated nuclei (∼400 ns), Figure 7d. The isolated cellular DNA studies show that complex **2** is highly efficient at labeling chromatin and chromosome structures both within live cells and naked DNA. A reduction in the lifetime was also observed in PLIM measurements performed on HeLa cells incubated with **2**, see Figure S15. Notably, a change in the lifetime distribution in the field of view (FOV) of two-dimensional (2D) confocal PLIM slices taken from three-dimensional (3D) imaging of **2**-labeled live HeLa cells was observed as the FOV moves through the cell. Above the midpoint of the cell, longer lifetimes (ca. 800 ns) characteristic of localization in the cytoplasm are observed and dominant, Figure S17a; while at the midpoint of the cell, the PLIM signal shows the presence of lifetimes characteristic of location in the cytoplasm (∼800 ns) and quenching in the nucleus (<600 ns). This is shown to have a greater effect in the distributions selected for the specific regions of interest, see Figure S16. To further explore the uptake, a localization study was performed on Hela cells by staining the endoplasmic reticulum (ER), lysosomes, and the nucleus. PLIM images recorded using a long-pass filter show the localization of (**2**) in the cell, see Figure S17a,b, while the lysotracker and nuclear stain are visible with the short-pass filter in place (Figure S17c,d). A significant overlap in the signals is observed, which indicates that the complex is found in both of these subcellular compartments. Similarly, combined staining indicates that **2** is also located in the ER, see Figure S18. While the complex is found throughout the cell, its presence in the nucleus is of interest as this indicates the potential of these probes to trigger DNA photodamage.

## 4 Conclusions

In this study, we described the distinct properties of two new Ru(II) polypyridyl complexes comprising extended polypyridyl ligands that are structural isomers. This work highlights the significant impact the connectivity of the aromatic network of intercalating polypyridyl ligands has on the photophysical properties. The highly luminescent **2** is dominated by the TAP-based MLCT, while the more weakly luminescent **1** is dominated by the formation of the ^3^ππ state. Both **1** and **2** bind strongly to DNA with the quenching of photoluminescence, which is found to occur by different mechanisms. Cell studies of the luminescent **2** reveal that localization in the nucleus is accompanied by a reduction in the luminescent lifetime, attributed to quenching by DNA. The complexes studied here have potential to act as photothermal agents through mechanisms that occur on vastly different time scales. In the case of **1**, the generation of the excited state in the intercalated complex does not result in markers of initial DNA damage on the ns time scale. In contrast, rapid formation of the guanine radical cation is observed for **2**. Previous studies have confirmed the role of the long-lived ^3^ππ state in photocleavage of DNA through single oxygen generation.^6,8^ The results of this study have implications for the development of PDT agents by showing that a slight change in the ligand structure results in a dramatic change in the mode of action of the complexes where DNA damage can be expected to occur through direct electron abstraction from guanine for **2** while in the case of **1** singlet oxygen generation will be the likely mode of action. Future studies will probe the ability of the enantiomers of these extended ruthenium polypyridyl complexes to bind to G-quadruplex forming structures and employ super resolution studies such as expansion microscopy or STED together with PLIM to elucidate any preference of the probe to specific DNA structural conformations and the role and efficiency of both mechanisms to cause DNA photodamage.

## Supporting Information Available

Additional experimental details, materials, and methods, including TrA and TRIR data, spectroscopic data from titrations and additional images from cellular studies (PDF)
