# Supplementary material for: Contrasting Photosensitized Processes of Ru(II) Polypyridyl Structural Isomers Containing Linear and Hooked Intercalating Ligands Bound to Guanine-Rich DNA
Source: J Phys Chem B. 2024 Aug 6;128(32):7803–12. doi: 10.1021/acs.jpcb.4c04129 (PMC11331526; doi:10.1021/acs.jpcb.4c04129)
Supplement: Supplementary file 1 — jp4c04129_si_001.pdf [file jp4c04129_si_001.pdf]

Supporting Information

Contrasting Photosensitised Processes of Ru(II) Polypyridyl Structural Isomers Containing Linear and Hooked Intercalating Ligands Bound to Guanine-Rich DNA

Mark Stitch,[a] Rosie Sanders,[b] Igor V. Sazanovich[b] Michael Towrie[b], Stanley W. Botchway[b] and Susan J. Quinn[a*]

[a]*School of Chemistry, University College Dublin, Dublin 4, D04 V1W8, Ireland.*

[b]*Central Laser Facility, Research Complex at Harwell, STFC Rutherford Appleton Laboratory, Harwell Science and Innovation Campus, Didcot, Oxfordshire OX11 0QX, U.K.*

Table of contents

S1 Experimental S-2

S2 Figures and Schemes S-5

S3 References S-23

S1 Experimental

Chiral Resolution of [Ru(TAP)$_2$(dppn)] and [Ru(TAP)$_2$(bdppz)]

The column was loaded with a slurry of cation exchange CM Sephadex C-25 (GE healthcare), previously soaked overnight in water and then thoroughly degassed for 1 h, and allowed to settle overnight before passing water through the column for 2 h. A solution of (10 mg) the racemic [$\mathbf{1}^{2+}$][Cl]$_2$ and [$\mathbf{2}^{2+}$][Cl]$_2$ was dissolved in a 10 mL water prior to loading onto the Sephadex. Nanopure water was passed through the column until the complex was adsorbed fully onto the Sephadex and the water above was colourless. Additional CM Sephadex C-25 slurry was then added to top of the column to protect the loading bed from being disturbed and water was further passed through the column for 1 h to ensure all the complex was electrostatically bound. The mobile phase was then changed to aqueous (−)-O,O′-dibenzoyl-L-tartrate (Sigma-Aldrich) (0.1 M) and the pump speed was gradually increased to 1 mL min^{-1}. The column was kept in darkness when being used. Additional Sephadex slurry was added during the running of the column to maintain the height of the Sephadex, as the increased ionic strength of the mobile phase caused the Sephadex to shrink during the resolution. During recycling, the speed of the pump was reduced to 0.1 mL min^{-1}. Clear separate bands were observed with the lambda enantiomer eluting first. The enantiopure species were isolated by gentle shaking with amberlite beads for a period of 8 h, the removal of tartrate was monitored via UV vis spectroscopy. The solvent was then removed under a reduced pressure and the complex was dissolved in MeCN and left in the fridge overnight to allow precipitation of the excess NaCl salt, which was removed by filtration. The enantiomers were further collected as their PF_6^- salt using a concentrated solution of NH_4PF_6. The precipitate was collected via centrifugation and the precipitate was further washed with water 3 times. The chloride form was regenerated by shaking in methanol with amberlite IRA 402 (Sigma-Aldrich) beads at 5°C. The enantiomeric purity of the fractions was then analysed using circular dichroism and the Δε were recorded to ensure enantiomeric purity.

Time Resolved Spectroscopy: TRIR spectroscopy measurements were conducted on the ULTRA and LIFETIME apparatus at the Central Laser Facility (STFC Rutherford Appleton Laboratory, Harwell, UK). During the experiments the samples were raster scanned in the x and y directions to minimise photo-damage and re-excitation effects. The samples were excited at 400 nm for the Ru complexes. For the acquisition of the spectra, the polarisation of the pump pulses at the sample were at the “magic” angle relative to the probe and was set to 1 µJ for TRIR measurements and to between 400 nJ and 500 nJ for TrA measurements.

ps time domain measurements (ULTRA apparatus): Both pump and probe pulses were generated by the ULTRA laser system, comprising of a dual output titanium sapphire chirped pulse amplifier (Thales Laser), producing an 800 nm (0.8 mJ) output with 40 fs pulse duration and a repetition rate

of 10 kHz, which is split to generate the pump and probe pulses. The pump pulses (1 μJ, 50 fs pulse duration) for ps-TRIR and ps-TrA experiments were generated by second (400 nm) harmonic generation in a β-barium borate (BBO) crystal.

To generate the mid-infrared probe light for TRIR measurements, the 800 nm output was used to pump a white-light continuum-seeded optical parametric amplifier (OPA) (Light Conversion, TOPAS). The generated signal/idler outputs were then difference frequency mixed by an $AgGaS_2$ crystal producing output pulses of *ca*. 400 cm^{-1} bandwidth in the mid-infrared region of the spectrum with *ca.* 50 fs pulse duration. To further increase the signal-to-noise ratio for the TRIR measurement, the IR probe beam was further split by a germanium beamsplitter, with one beam forming the reference probe, detected by a 64-pixel mercury cadmium telluride (MCT, IR Associates) detector, this reference probe allows constant monitoring and correction for fluctuations in the probe spectrum and intensity during data acquisition. The second beam is focused on to the sample and recorded on two 128-pixel MCT detectors. The output of the two 128-pixel detectors was then spliced during data processing to create a single compete spectrum.

The probe pulse for TrA measurements is generated by focusing part the 800 nm output beam through a white-light continuum generator (CaF_2 plates). This white light is then dispersed through a grating monochromator after passing through a 400 nm notch filter, to remove scatter from the excitation beam. The TrA spectrum was then recorded using a 512-pixel silicon detector (Quantum Detectors). The delay between pump and probe pulses in these experiments was achieved using an optical delay line.

ps to ms time domain experiments: The Time-Resolved Infrared (TRIR) measurements on the time range from ps to ms were performed on LIFEtime apparatus at the STFC Central Laser Facility.[1] The LIFEtime setup is driven by the two Yb:KGW amplifiers (PHAROS, Light Conversion, each amplifier operating at 100 kHz). The two amplifiers are optically synchronised by sharing the same oscillator operating at 80 MHz repetition rate. One amplifier is driving a single 515 nm pumped OPA (ORPHEUS HP, Light Conversion) which is capable to generate the output beam across 210 – 2600 nm range which used as a pump source in TRIR experiment. The pulse picker built into each PHAROS amplifier provides a simple way to adjust the repetition rate of pump pulses, which was set to 2 kHz in the present work. The wavelength of the pump beam was set to 400 nm in the present work, with the pulse energy at sample and the pump spot size to 500 nJ and 120 μm, respectively. The second amplifier was used to pump simultaneously two mid-IR OPA (ORPHEUS ONE and LYRA DFG unit, Light Conversion) generating two mid-IR probe beams for the TRIR experiment. The probing was performed at 100 kHz repetition rate. The pump and two probe beams were overlapped at the sample, and the two probe beams were subsequently sent to the home-built grating spectrographs and two individual probe spectra detected with 128-pixel MCT detectors each (IR Associates). The time delay

between the pump and the probe beams was achieved using the combination of the optical delay line (for the delay range fs to 12 ns), and oscillator round trip timing to achieve steps of 12 ns. The train of probe pulses coming at 100 kHz covers the time range from 10 μs and longer. The relative polarisations of the pump beam with respect to the two probes was set at “magic” angle. The sample was rastered in the X and Y to minimise sample degradation in the pump beam. The LIFEtime setup offers < 200 fs temporal resolution along with > 400 cm^{-1} combined spectral window from the two probe OPA’s.

All spectra were processed using the in-house Ultraview software provided by the Central Laser Facility (CLF). Spectra were calibrated from pixels into wavenumbers using the Ultracal software. The metal complex and DNA region (1250-1860 cm^{-1}) was calibrated by fitting to the absorption bands of polystyrene. All experiments were carried out at 298 K and samples were checked before and after the experiment by UV-Visible spectroscopy (PerkinElmer lambda 950 spectrophotometer) and FTIR in a Nicolet Avatar spectrometer.

S2 Figures and Schemes

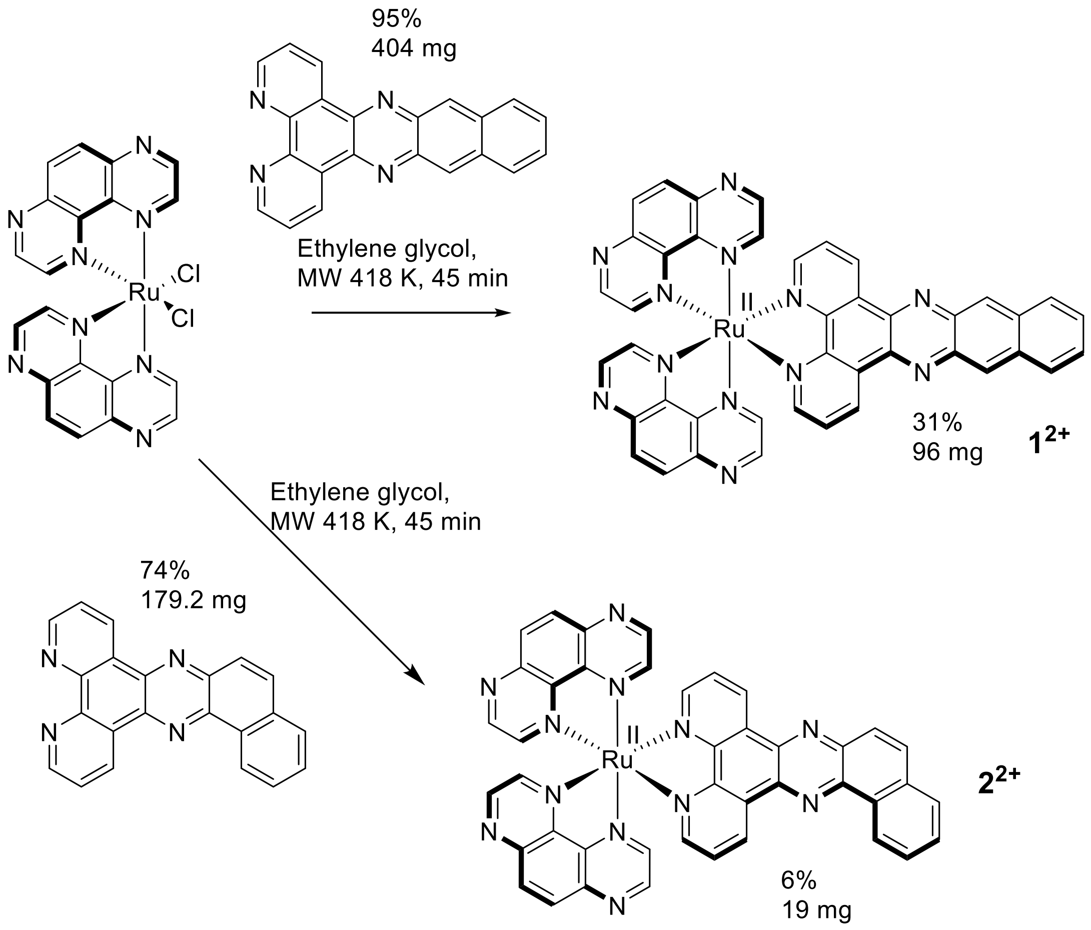

Scheme S1: Synthesis of $\mathbf{1}^{2+}$ and $\mathbf{2}^{2+}$ (prepared as both the chloride and PF_6^- salts).

(a)

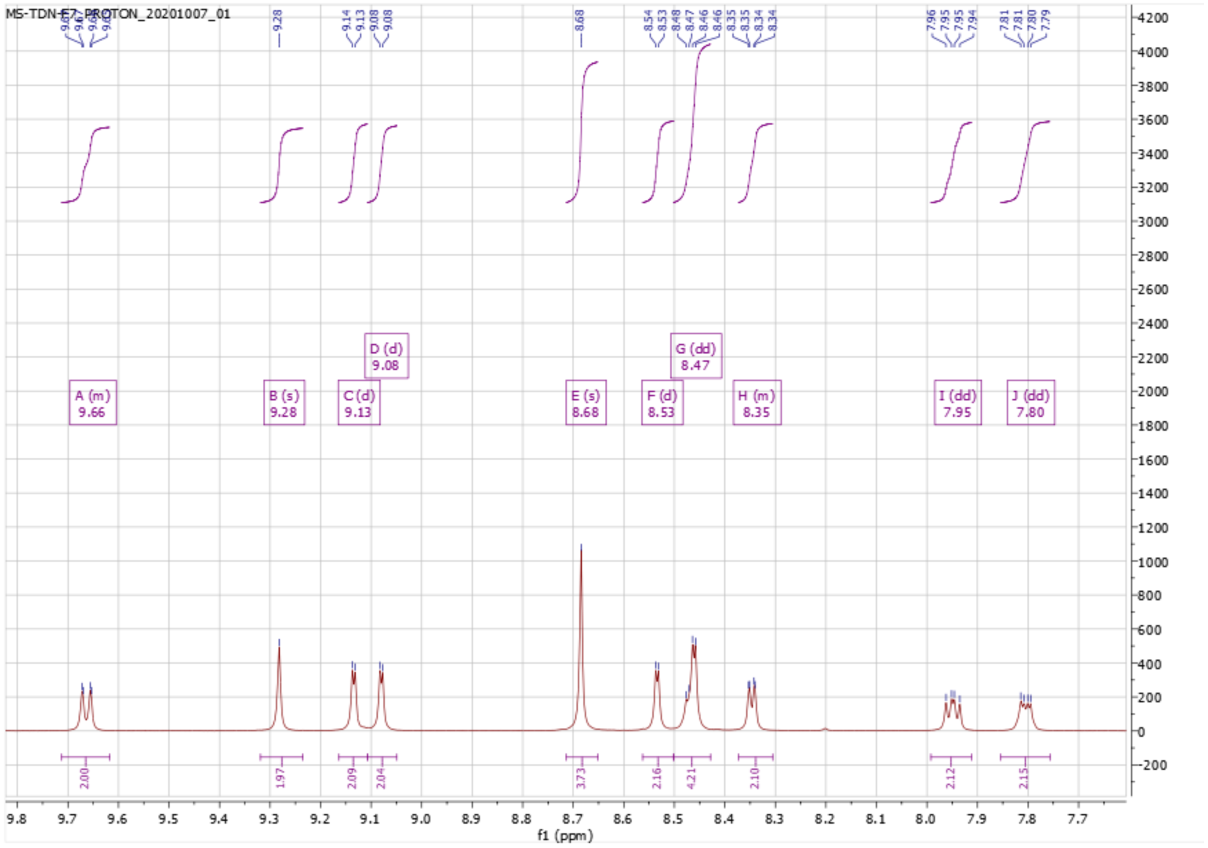

(b)

Spectrum Peaks

m/z	m/z (Calc)	Diff (ppm)	Abund	Height %	Height % (Calc)	Ion Species	Z
827.1000	827.1006	-0.69	836	14.10	11.53	M+[-Cl]	1
828.1067	828.1034	3.96	246	4.15	5.77	M+[-Cl]	1
829.0988	829.0993	-0.55	709	11.96	9.00	M+[-Cl]	1
830.0999	830.0992	0.75	2007	33.84	30.58	M+[-Cl]	1
831.0990	831.0988	0.27	2565	43.24	41.72	M+[-Cl]	1
832.0984	832.0989	-0.60	3713	62.59	61.17	M+[-Cl]	1
833.0983	833.0981	0.19	5931	100.00	100.00	M+[-Cl]	1
834.1009	834.0994	1.79	3243	54.68	54.46	M+[-Cl]	1
835.0981	835.0979	0.28	4298	72.46	75.48	M+[-Cl]	1
836.1007	836.1002	0.59	2167	36.53	32.89	M+[-Cl]	1
837.0987	837.0983	0.50	1423	24.00	20.15	M+[-Cl]	1
838.1001	838.0995	0.69	456	7.68	7.43	M+[-Cl]	1
839.1046	839.1017	3.43	89	1.51	1.67	M+[-Cl]	1

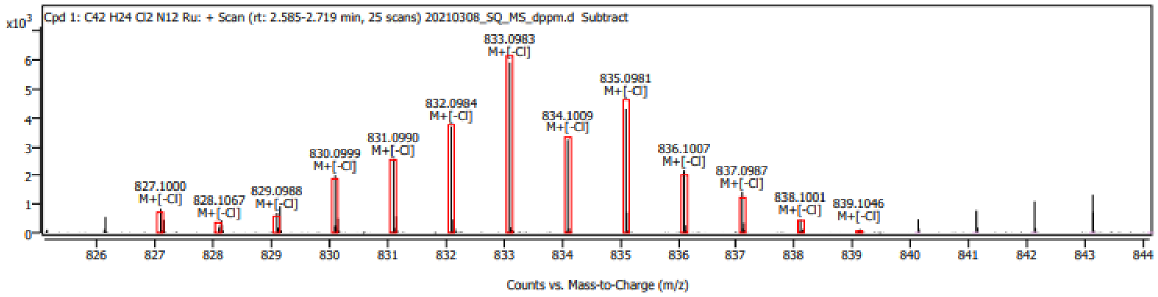

Figure S1: (a) ^{1}H NMR spectrum of [Ru(TAP)$_2$(dppn)].2Cl (400 MHz, D_2O) and (b) HRMS data for [Ru(TAP)$_2$(dppn)] ([M][Cl]$^+$).

(a)

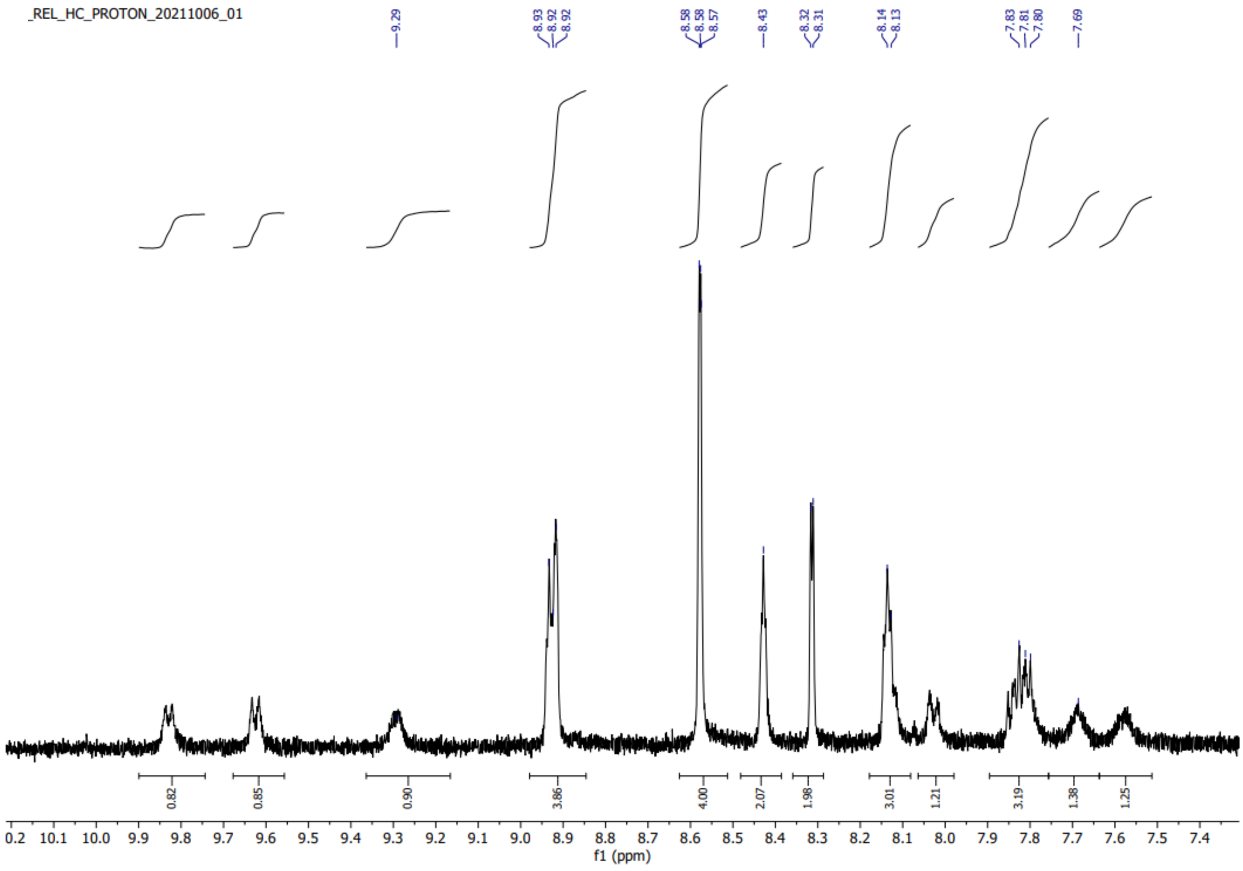

(b)

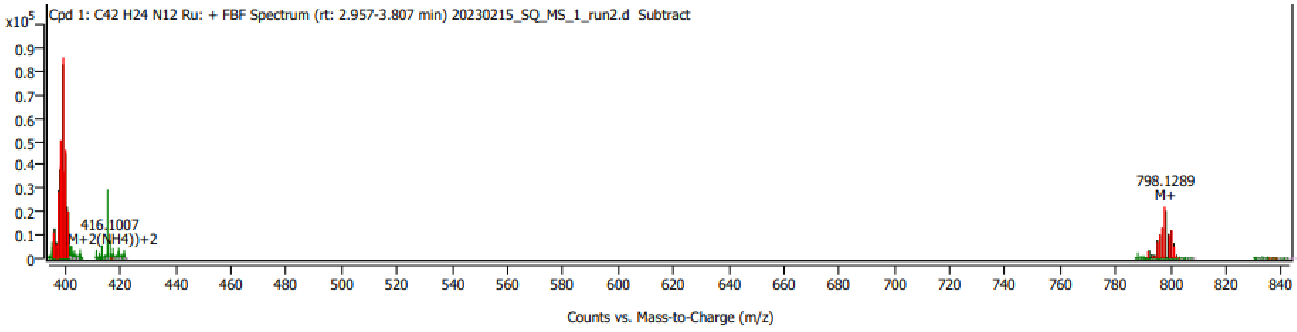

Figure S2: (a) ^{1}H NMR spectrum of [Ru(TAP)$_2$(bdppz)].2Cl (**2**) (400 MHz, D_2O) and (b) HRMS data for [Ru(TAP)$_2$(bdppz)] (**2**) ([M]$^+$).

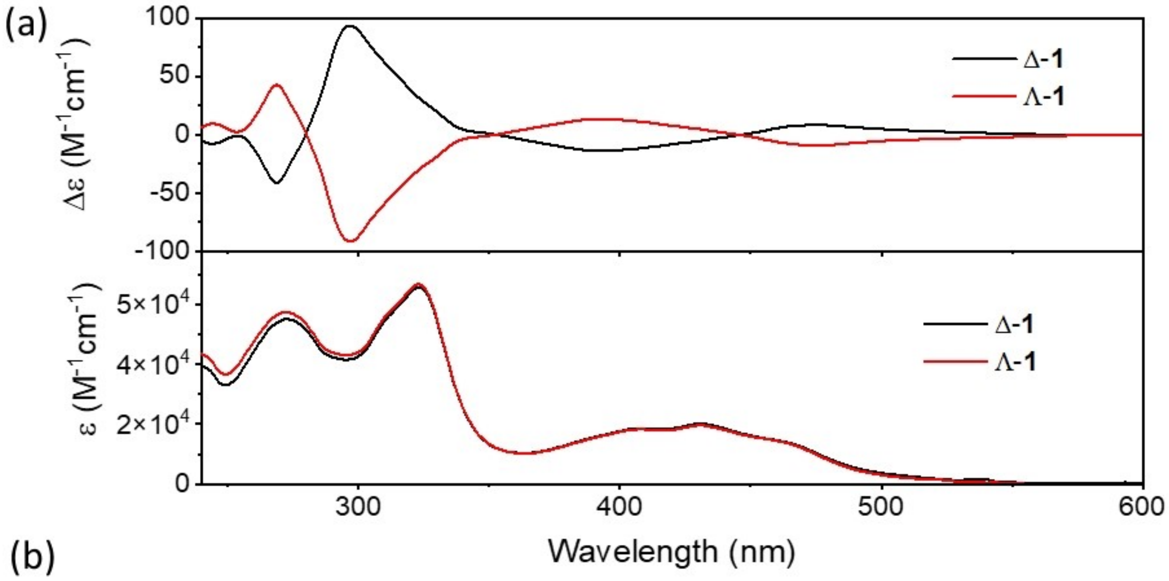

(b)

Δε M⁻¹ cm⁻¹	Δε(269 nm)	Δε(297 nm)	Δε(392 nm)	Δε(474 nm)
Δ-**1**	42	-92	13	-9
Λ-**1**	-42	93	-14	8

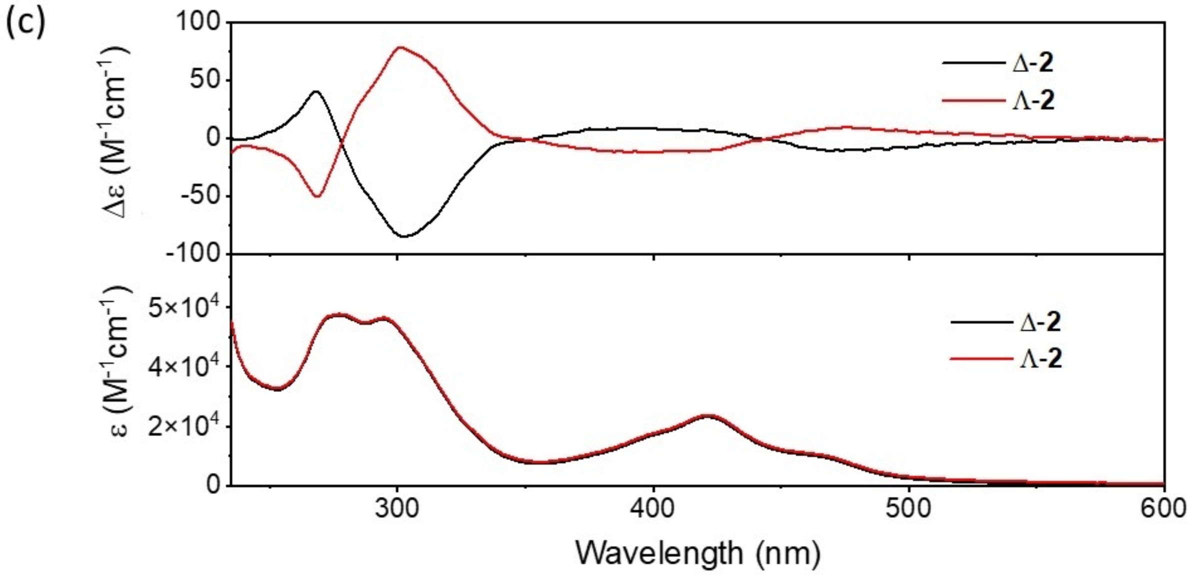

(d)

Δε M⁻¹ cm⁻¹	Δε(268 nm)	Δε(301 nm)	Δε(392 nm)	Δε(476 nm)
Δ-**2**	41	-84	9	-11
Λ-**2**	-49	78	-11	10

Figure S3: (a) UV-Vis spectrum of the Δ-1 and Λ-1 enantiomers in water and CD spectrum of the enantiomers (b) summary of the optical properties. (c) UV-Vis spectrum of the Δ-2 and Λ-2 enantiomers in water and CD spectrum of the enantiomers (d) summary of the optical properties.

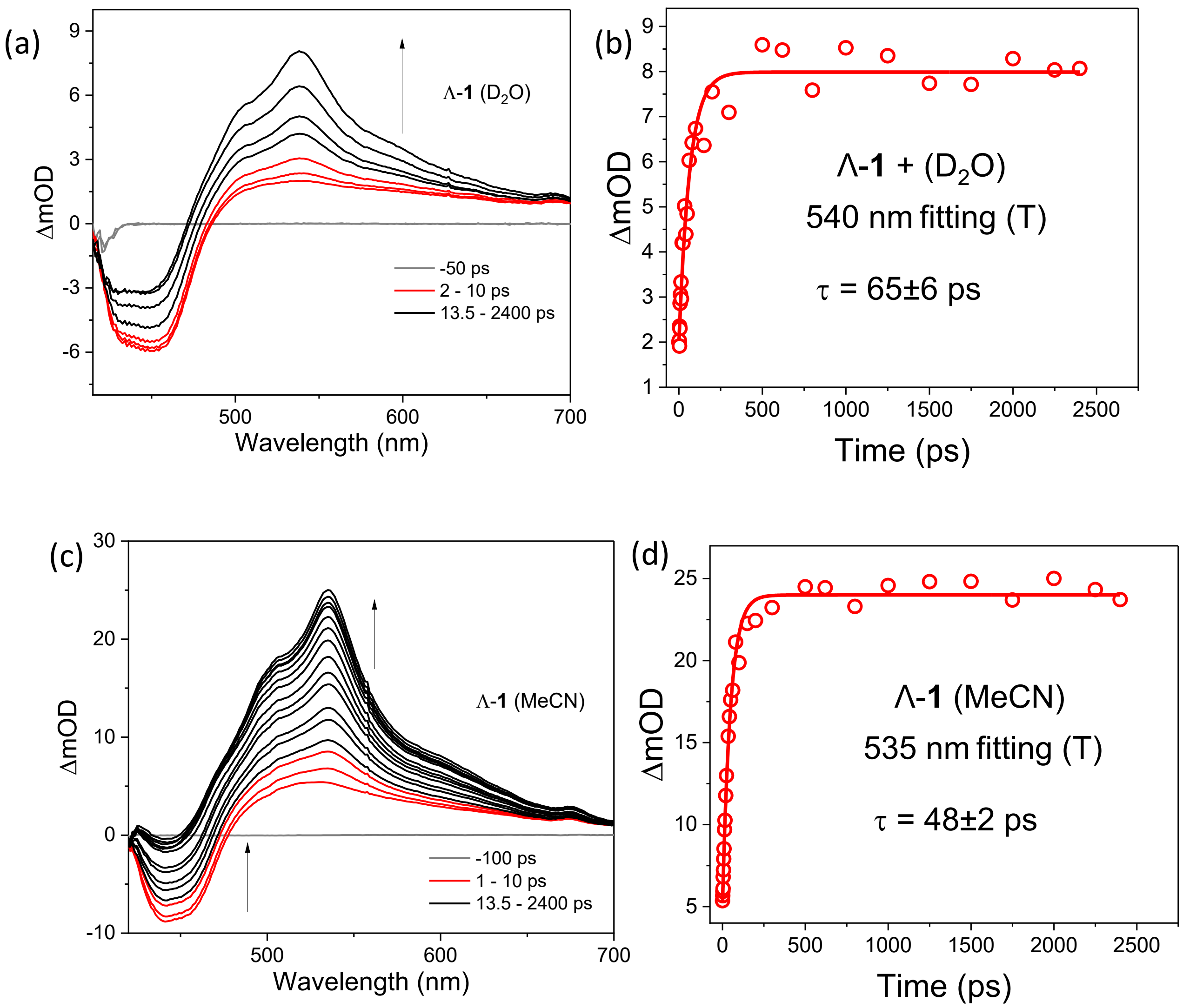

Figure S4: TrA spectra and accompanying kinetic fitting plots of Λ-[Ru(TAP)$_2$(dppn)] (Λ-1) (50 μM) in the presence of (a-b) D_2O and (c-d) MeCN (λ_{ex}= 400 nm, 400 nJ, 150 fs).

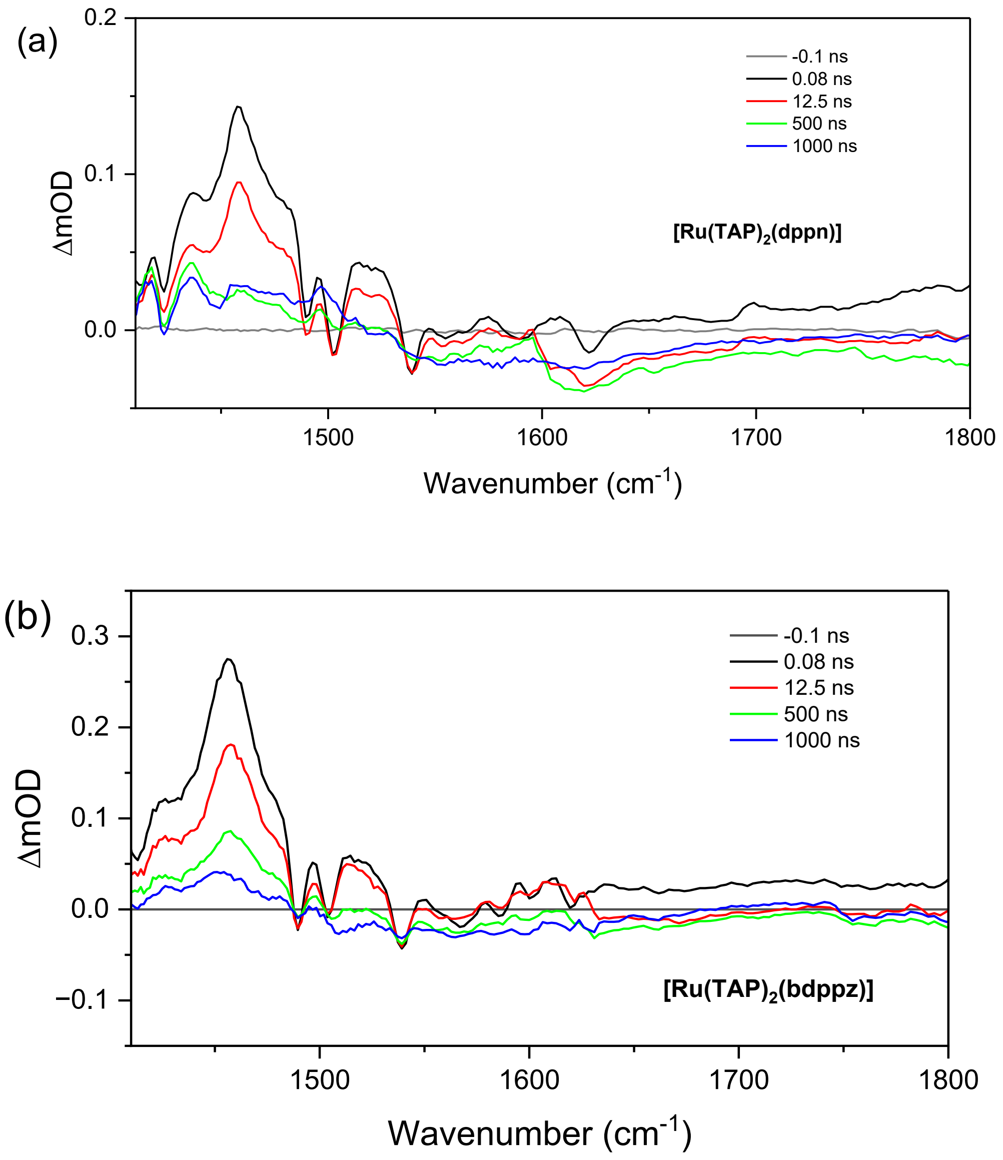

Figure S5: TRIR spectra of (a) Λ-[Ru(TAP)$_2$(dppn) (**Λ-1**) (0.2 mM) and (b) Λ-[Ru(TAP)$_2$(bdppz) (**Λ-2**) (0.2 mM) in 50 mM phosphate buffer D_2O (λ_{ex}= 400 nm, 2 kHz,150 fs).

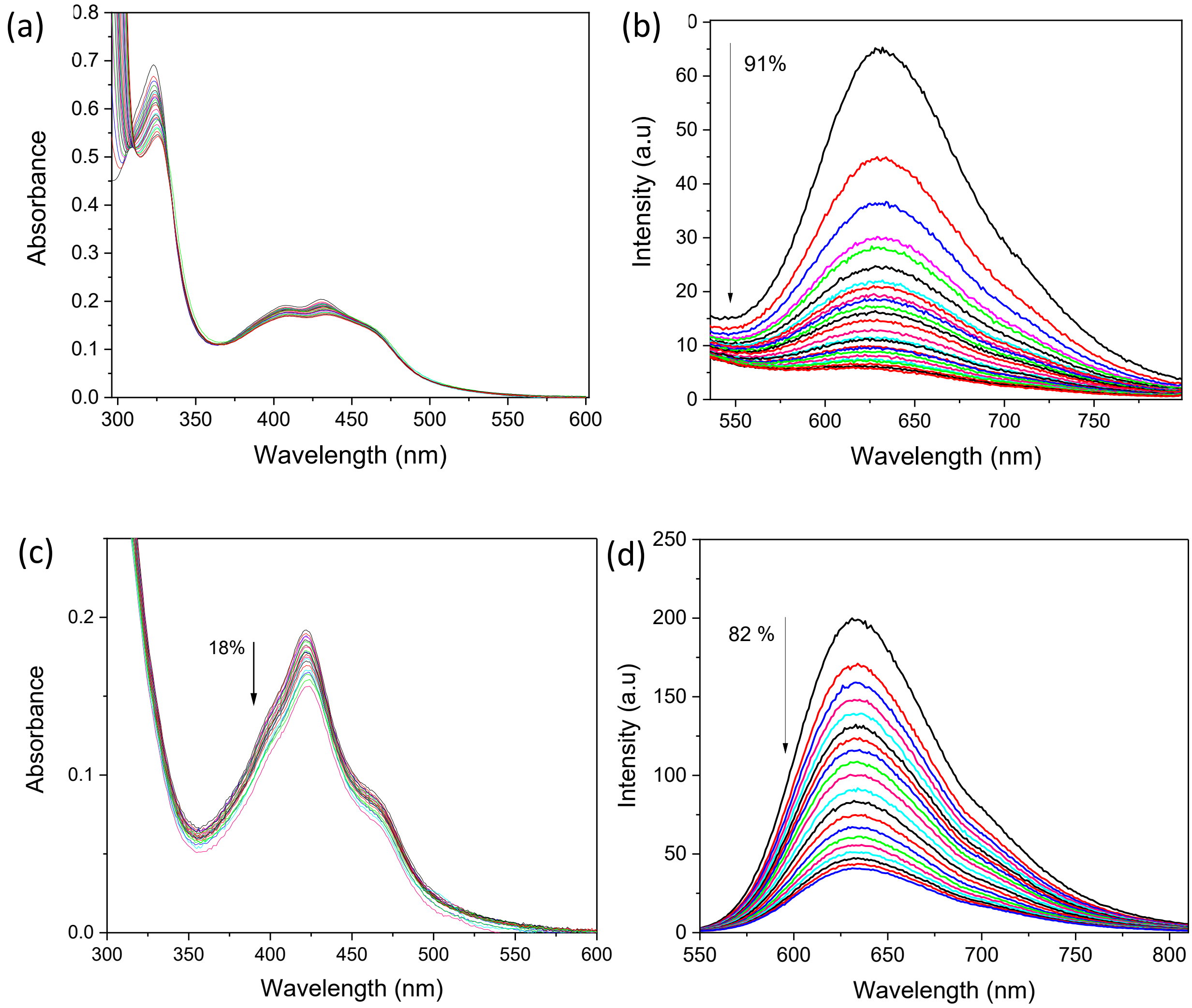

Figure S6: UV and luminescence spectra of (a-b) *rac*-[Ru(TAP)$_2$dppn]$^{2+}$ **1** (12.5 μM) and (c-d) *rac*-[Ru(TAP)$_2$bdppz]$^{2+}$ **2** (10.4 μM) titrated against increasing concentrations of (0 – 35 mM) GMP in water at pH 7.

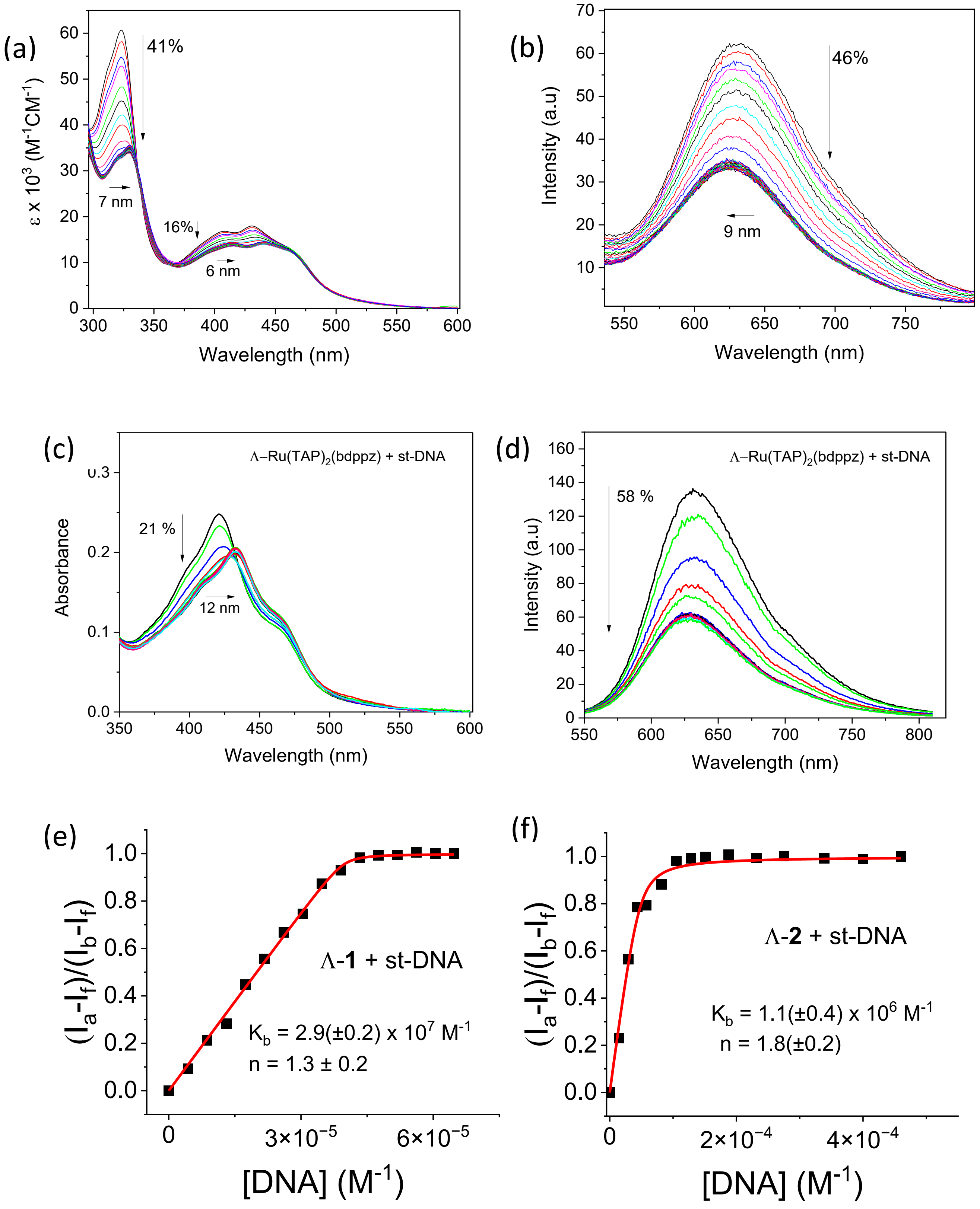

Figure S7: UV and luminescence spectra of (a,b) Λ-**1** (16.2 μM) and (c,d) Λ-**2** (13 μM) titrated against increasing concentrations of st-DNA (0 → 0.24mM) in 50 mM phosphate buffer at pH 7. Determination of the binding constant for (e) Λ-**1** and (f) Λ-**2** in the presence of st-DNA n 50 mM phosphate buffer. Plots of $(I_a - I_f)/(I_b - I_f)$ fitting at *ca.* 600 nm vs. [DNA] (per nucleobase) and non-linear curve fitting of the data (—) using the method of Bard et al.[2]

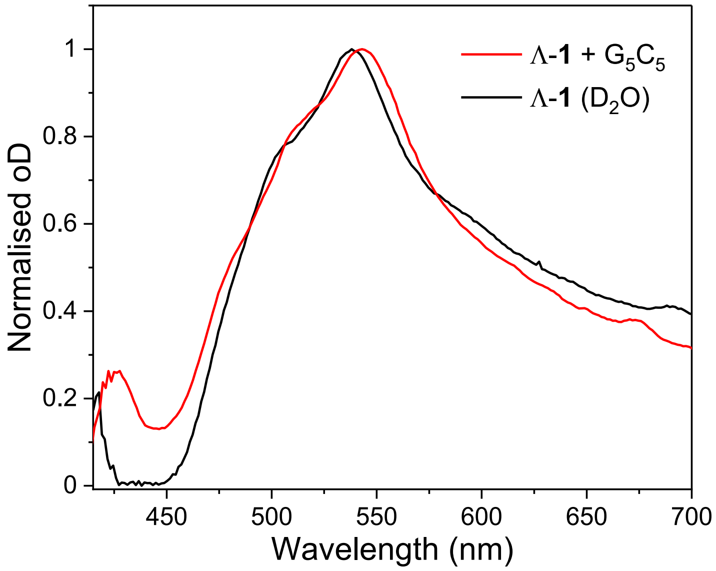

Figure S8: Comparative TrA spectroscopy of the normalised spectra recorded for 50 μM of Λ-**1** in D_2O and in the presence of $d(G_5C_5)_2$ DNA (75 μM double stranded) in 50 mM phosphate buffer D_2O (λ_{ex}= 400 nm, 400 nJ, 150 fs).

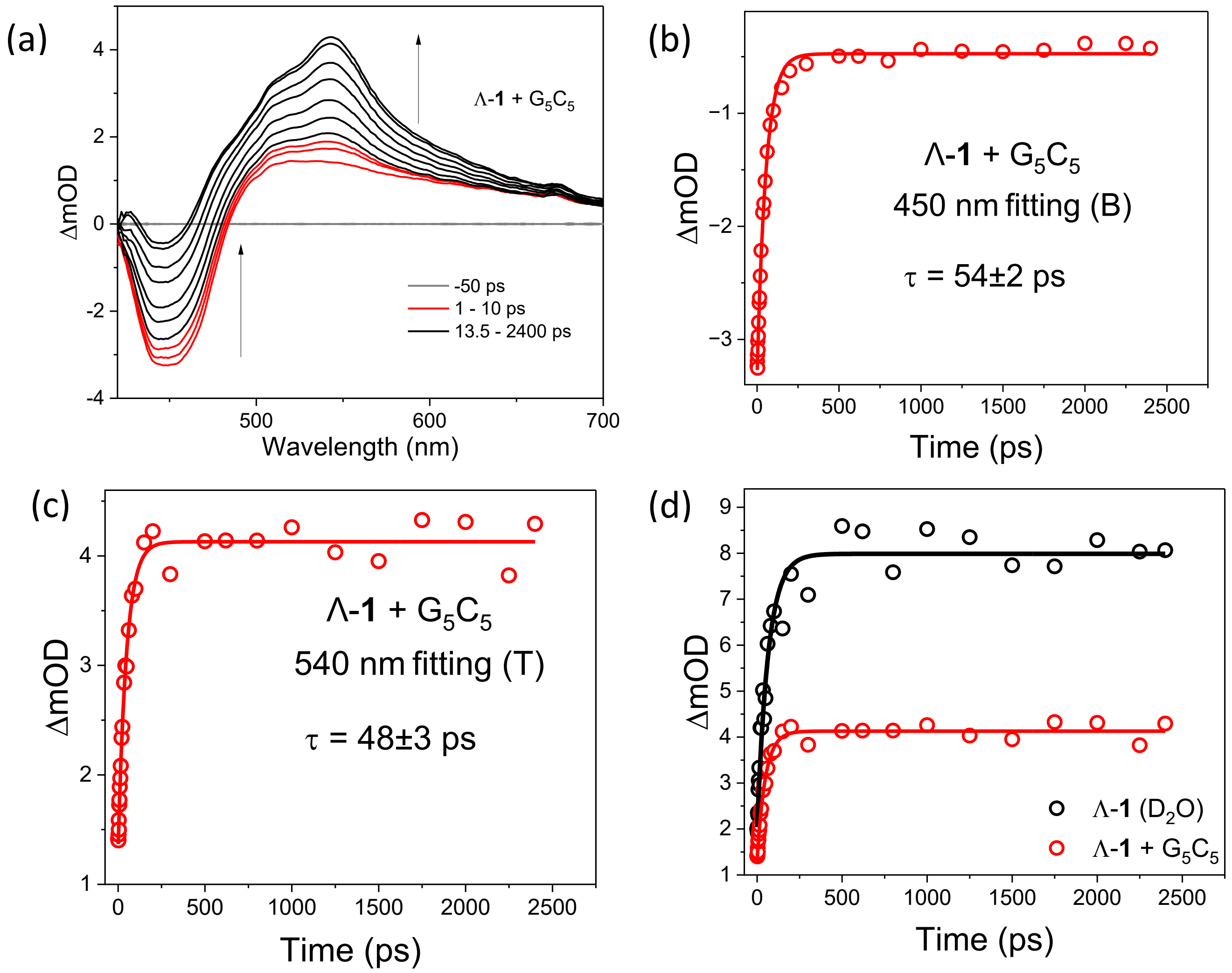

Figure S9: (a) TrA spectra of Λ-**1** (50 μM) in the presence of d(G_5C_5)$_2$ DNA (75 μM) in 50 mM phosphate buffer D_2O (λ_{ex}= 400 nm, 400 nJ, 150 fs). (b-c) Kinetic fitting plots of bleach and transient bands in the presence of G_5C_5 DNA (75 μM) in 50 mM phosphate buffer D_2O (λ_{ex}= 400 nm, 400 nJ, 150 fs). (d) Comparative kinetic plot of the evolution of the transient band (540 nm) in D_2O and d(G_5C_5)$_2$.

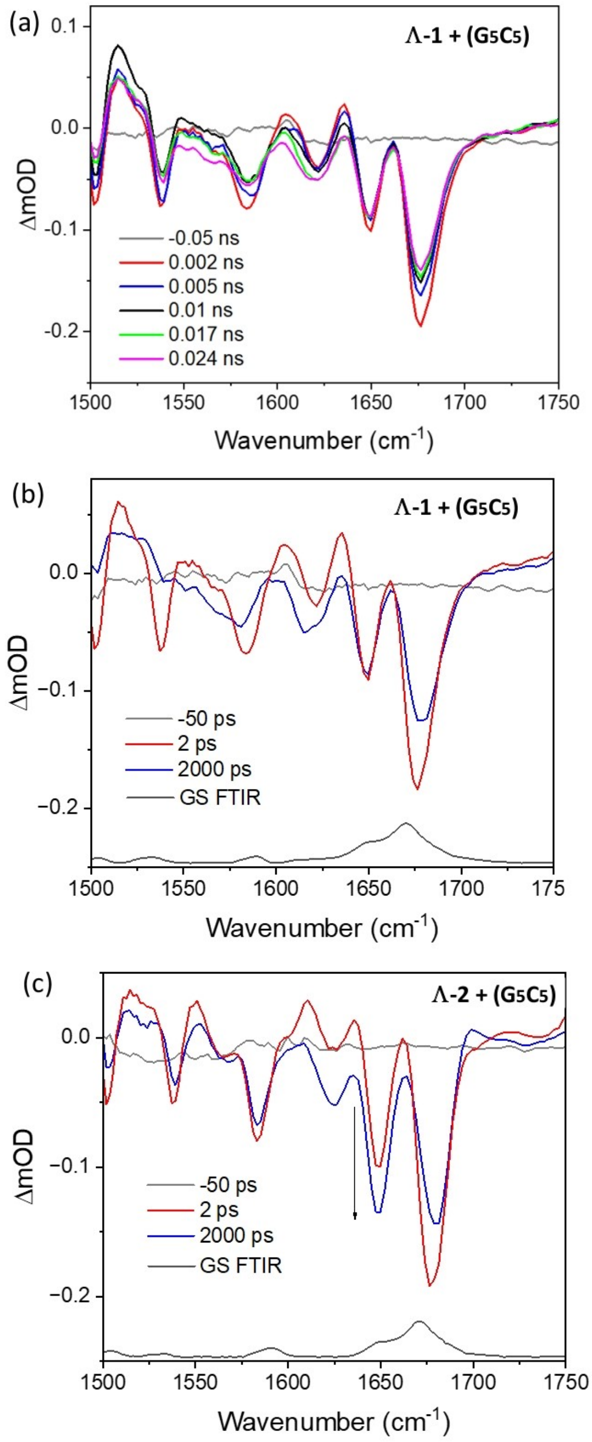

Figure S10: TRIR spectra of 200 μM Λ-**1** recorded in the presence of 300 μM d(G_5C_5)$_2$ (a) between 2 and 2000 ps and (b) at 2 ps and 2000 ps, 50 mM (λ_{ex}= 400 nm, 1 μJ, 150 fs). (c) 200 μM Λ-**2** recorded in the presence of 300 μM d(G_5C_5)$_2$ at 2 ps and 2000 ps, 50 mM (λ_{ex}= 400 nm, 1 μJ, 150 fs). All in 50 mM phosphate buffer (D_2O).

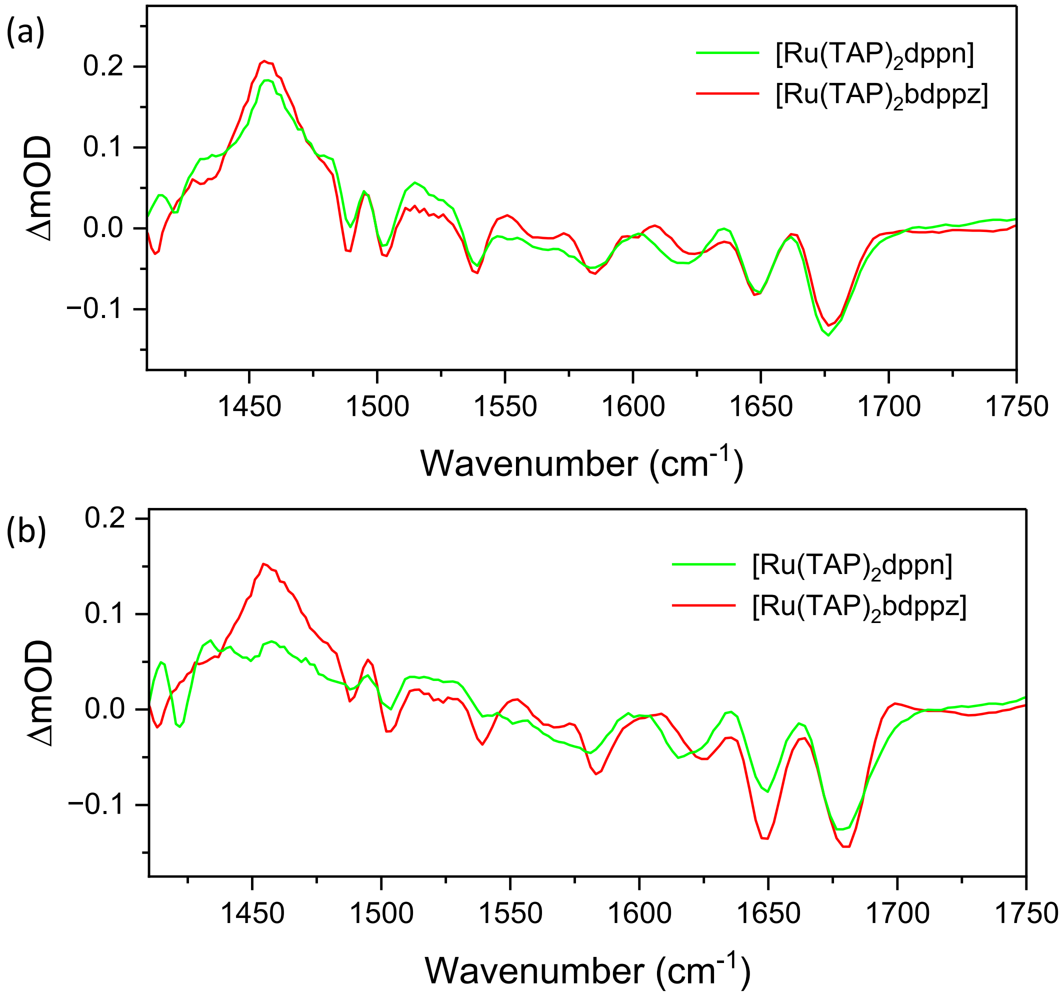

Figure S11: TRIR spectra overlay of 0.2 mM Λ-[Ru(TAP)$_2$(dppn (**Λ-1**) and Λ-[Ru(TAP)$_2$(bdppz) (Λ-**2**) in the presence of d(G_5C_5)$_2$ DNA (0.3 mM) (a) 24 ps after excitation and (b) 2000 ps after excitation (λ_{ex}= 400 nm, 2 kHz,150 fs).

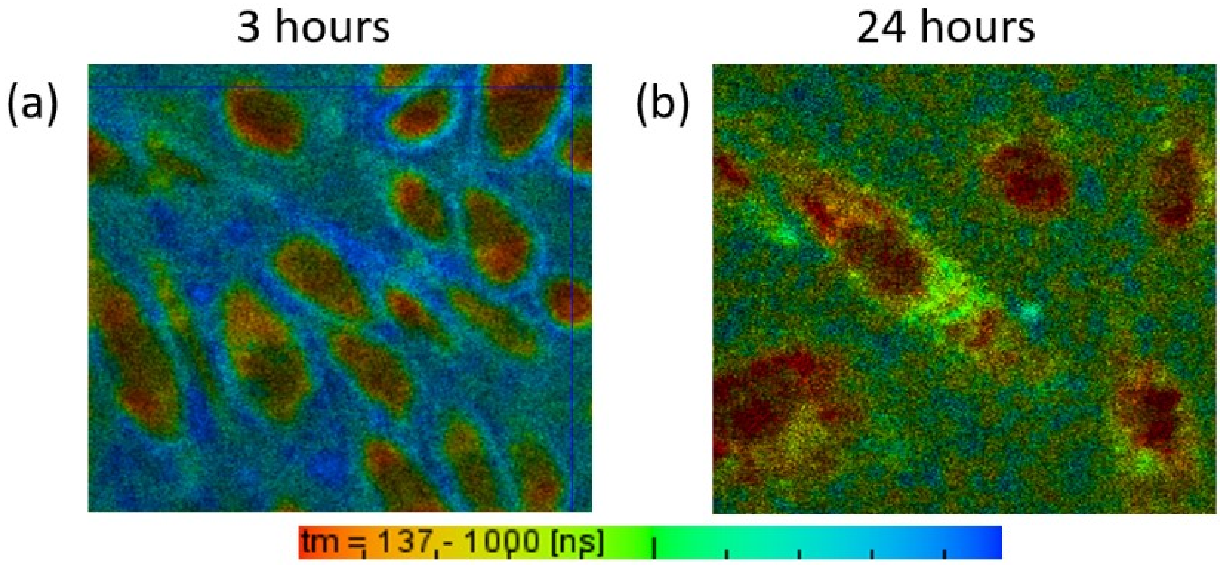

Figure S12. Live CHO cell imaging. PLIM channel (λ_{ex} = 405 nm/ $\lambda_{detection}$ = 450 nm long pass) for CHO cells incubated for (a) 3 hr and (100 μm region) (b) 24 hr with 50 μM of **2** and 60 x optical zoom (80 μm region).

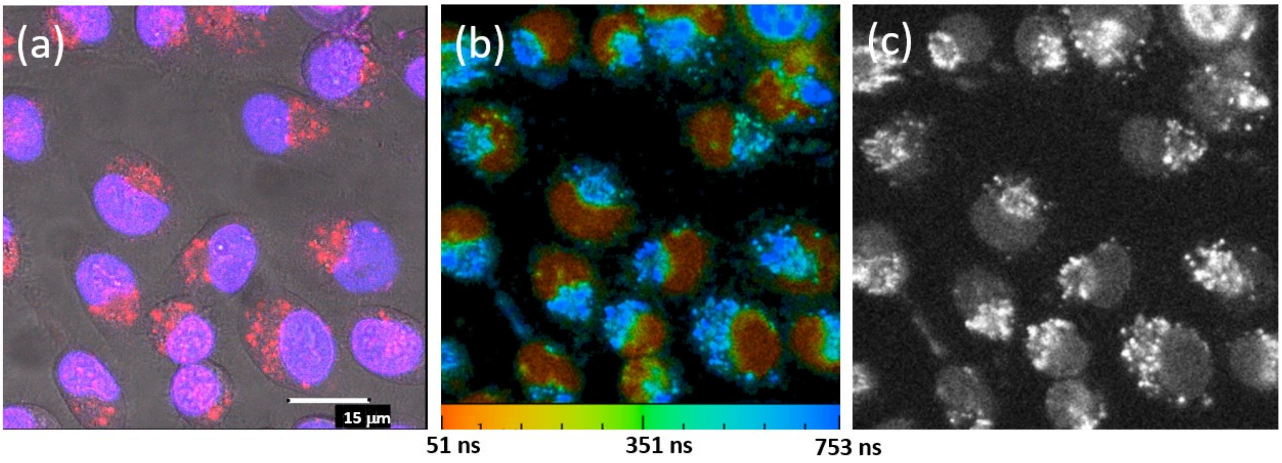

Figure S13: Steady state confocal images of live CHO cells labelled with DAPI and complex **2**. Excitation at 405 nm, emission in blue channel (Hoechst stain) and red channel (long pass 650 nm) (a) Confocal overlay of transmission light, DAPI and complex **2** channels. (b)-(c) PLIM images of the same field of view of **2** and CHO cells after 24-hour incubation period. DAPI was allowed to label for only 15 min.

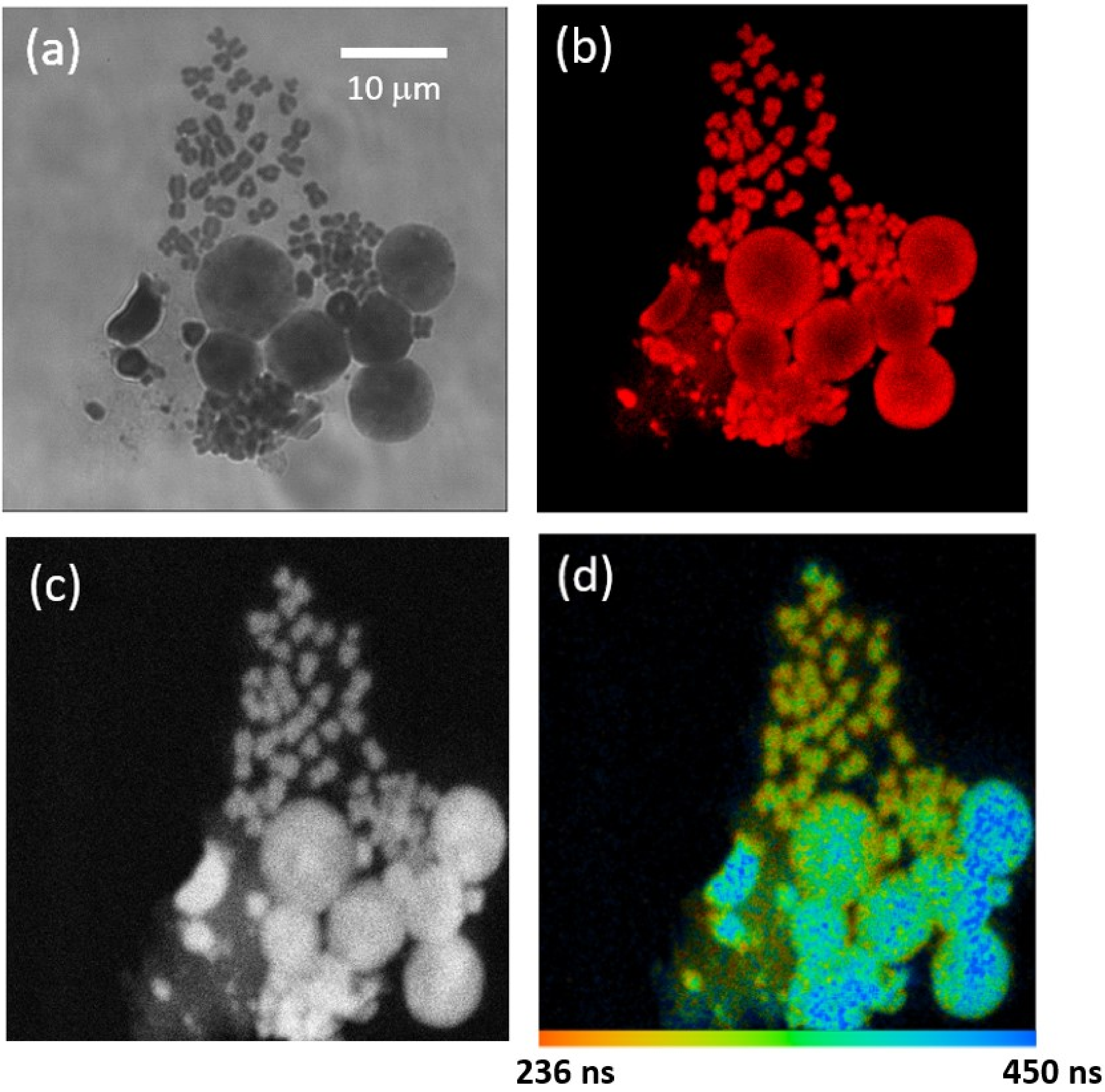

Figure S14 confocal and PLIM imaging of isolated nuclei and chromosomes incubated with complex **2** at room temperature. (a) and (b) confocal image of isolated nuclei and chromosomes incubated for 24 hr with 50 μM of **2** (60 x optical zoom) (c) and (d) PLIM channel (λ_{ex} = 405 nm/ $\lambda_{detection}$ = 450 nm long pass).

2D slices	Z-position	Lifetime distributions of whole FOV
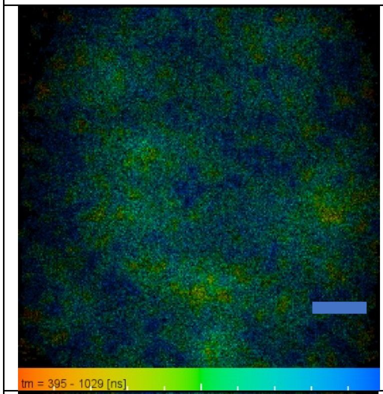	**A**<br>3 µm	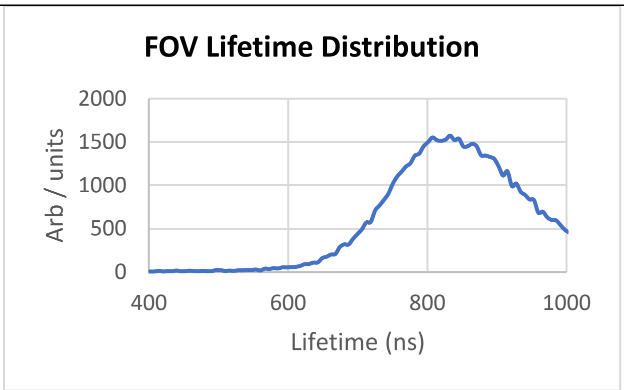
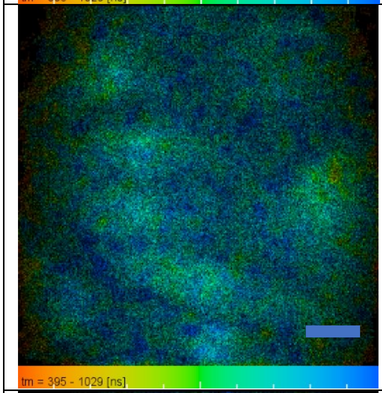	**B**<br>2 µm	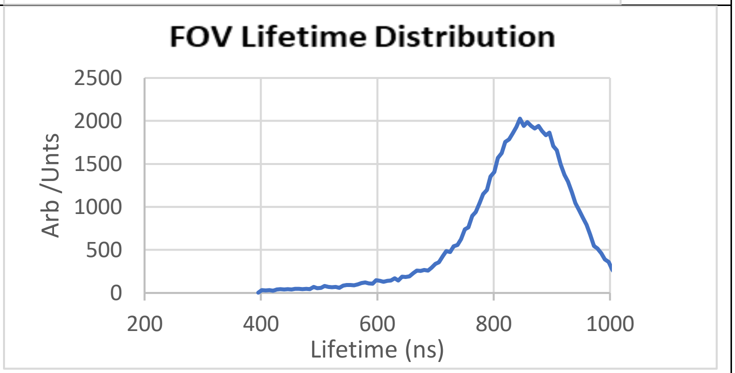
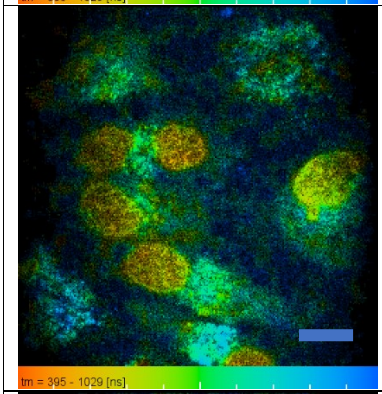	**C**<br>1 µm	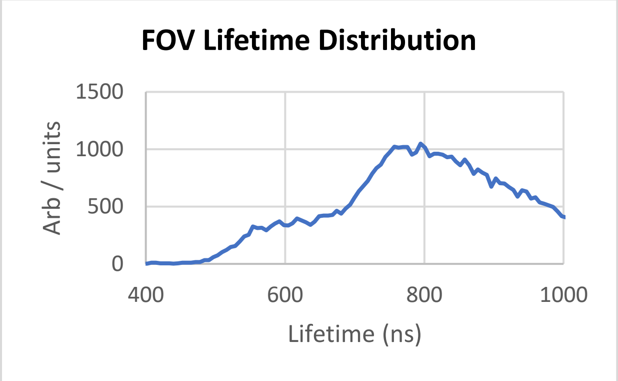
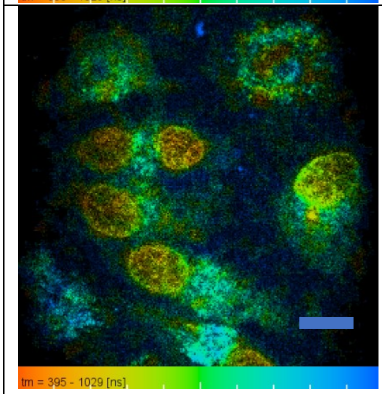	**D**<br>0 µm	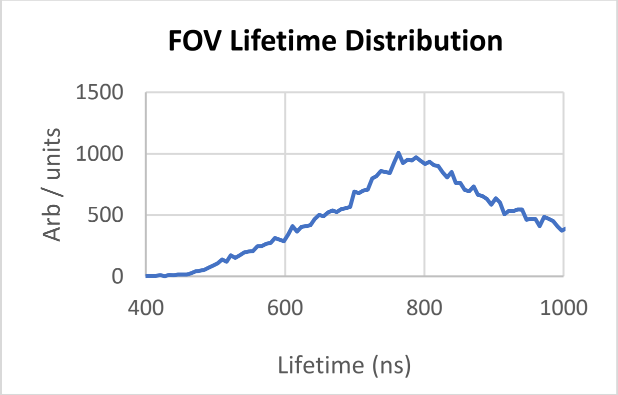

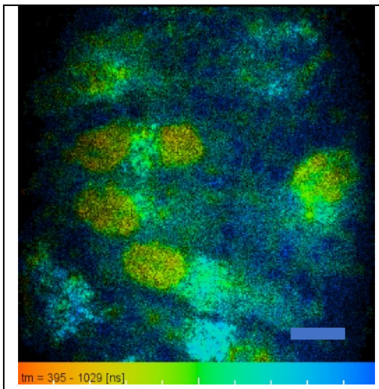	**E**<br>-1 μm	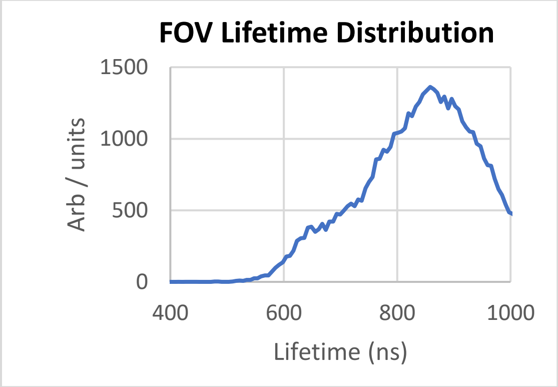
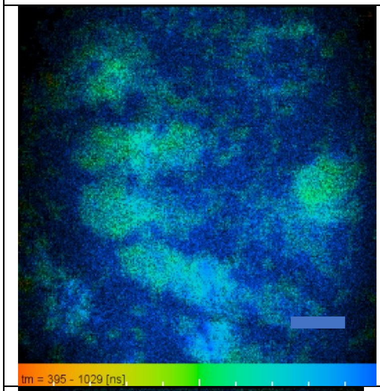	**F**<br>-2 μm	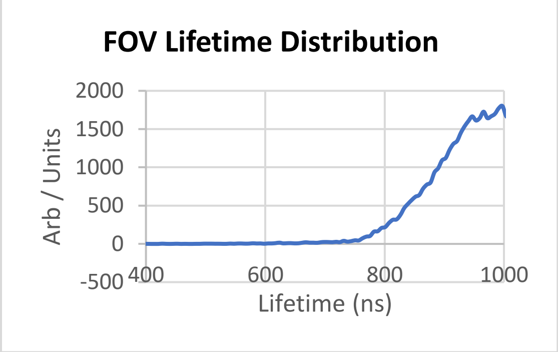
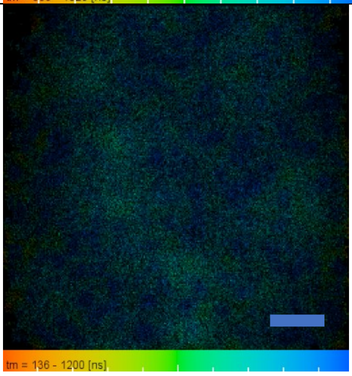	**G**<br>-3 μm	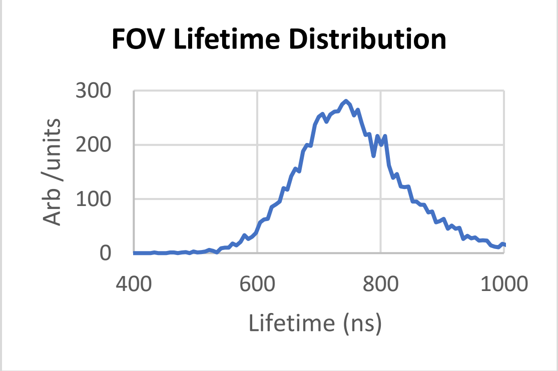

Figure S15. 2D confocal PLIM slices from 3D imaging of **2** labelled live HeLa cells. 405 nm excitation 610 nm long pass filter. 100 x oil immersion microscope objective, NA 1.49, at room temperature. Panel A- 3 μm above mid-point of the cells, indicating mostly cytoplasm and beyond into solution with no compound. Lifetime of ~800 ns indicative of absence of nuclear compound. B- 2 μm above mid-point of cells, similar lifetime to A, as little nuclear compound presence. C, D &E, mid-point of cells. PLIM signal shows the presence of both quenched nuclear lifetime (<600 ns) and that of cytoplasm (~800 ns). Panel F shows PLIM of cells 2 μm below the mid-point, again mostly cytoplasm as well as glass surface. PLIM value of >800 ns. Note, last slide, -3 μm, low photon counts, possible glass autofluorescence contribution hence should be ignored. Scale bar 20 μm.

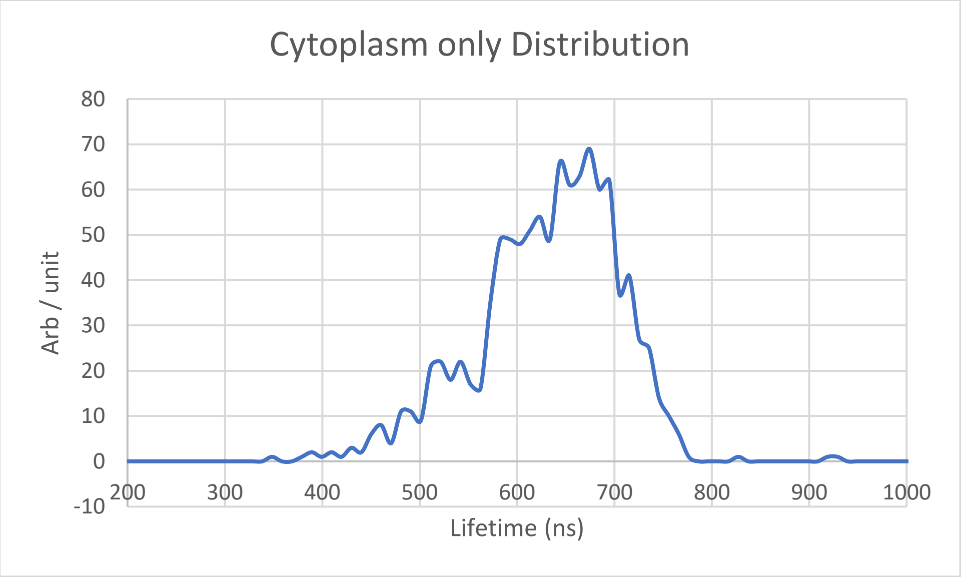

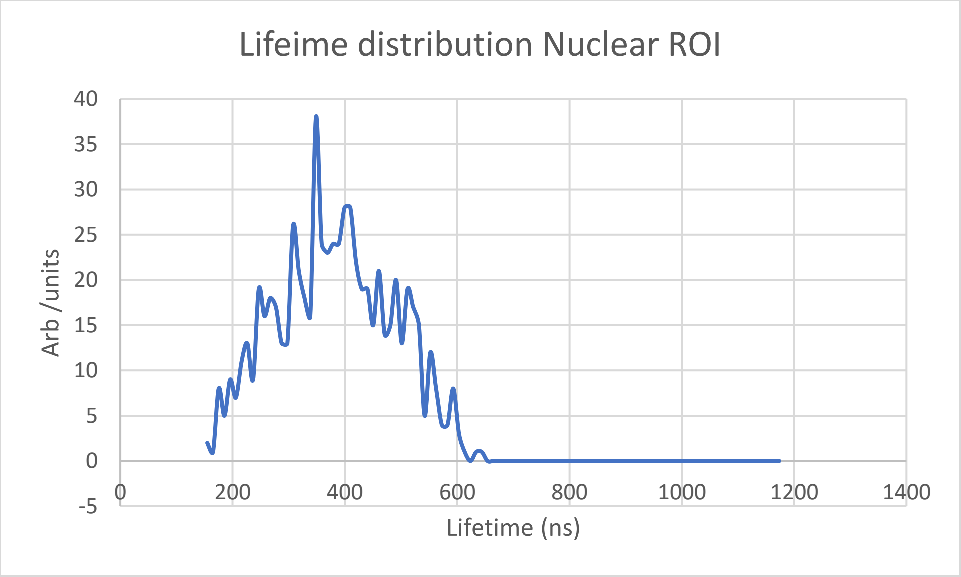

Figure S16. Region of interest (ROI) lifetime distributions extracted from mid-point of cell recorded in a 2D confocal PLIM slice from 3D imaging (S15, D) of **2** labelled live HeLa cells. 405 nm excitation 610 nm long pass filter.

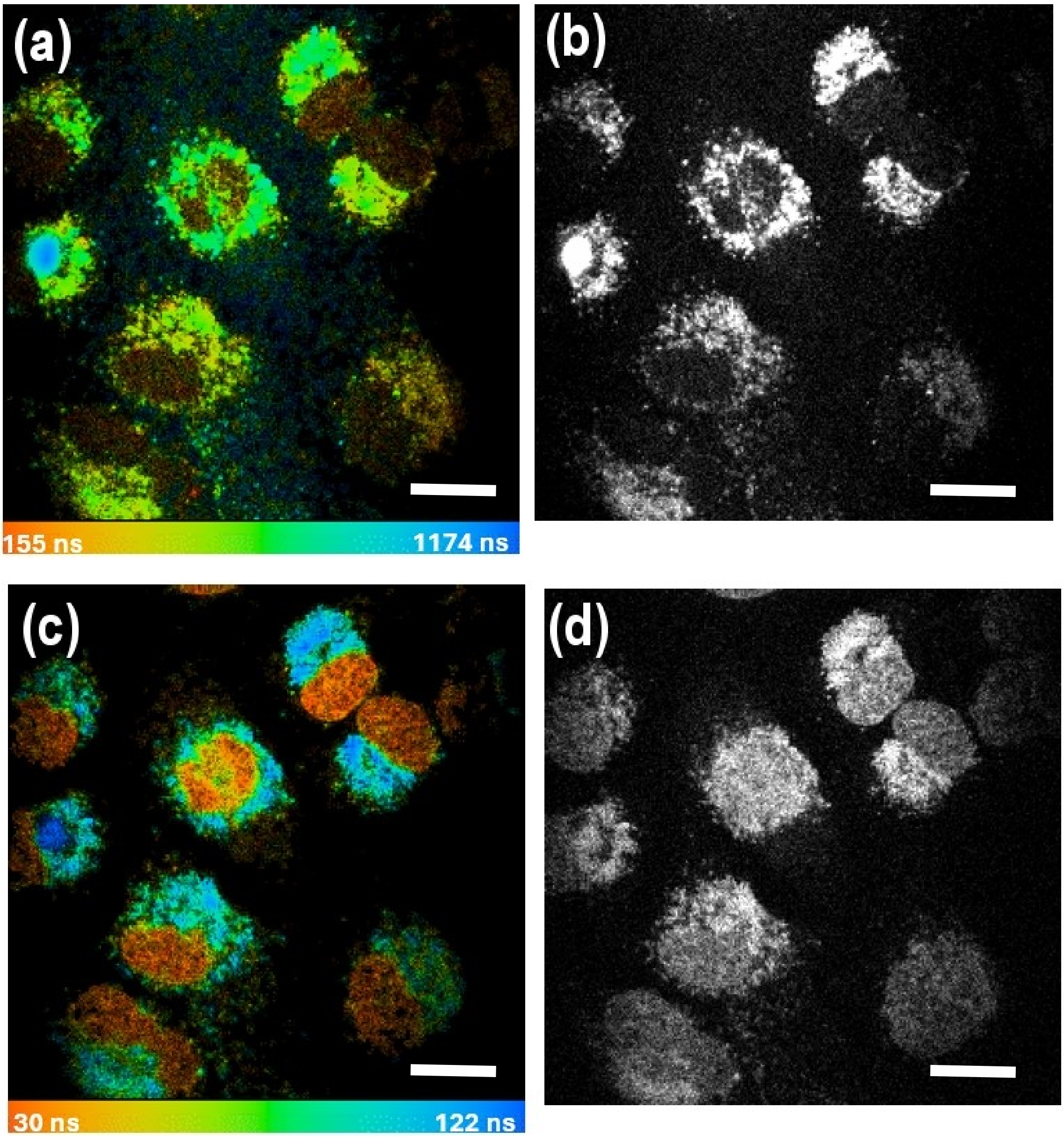

Figure S17: PLIM imaging of live HeLa cells incubated for 2 hr with 60 μM of **2** for 24hr followed by Hoechst and lysosome co-staining, (100 x oil immersion microscope objective, NA 1.49) at room temperature. (a) and (b) PLIM channel (λ_{ex} = 405 nm/ $\lambda_{detection}$ = 600 nm long pass), and (c) and (d) PLIM channel (λ_{ex} = 405 nm/ $\lambda_{detection}$ = 485 nm short pass). Scale bar 18 μm.

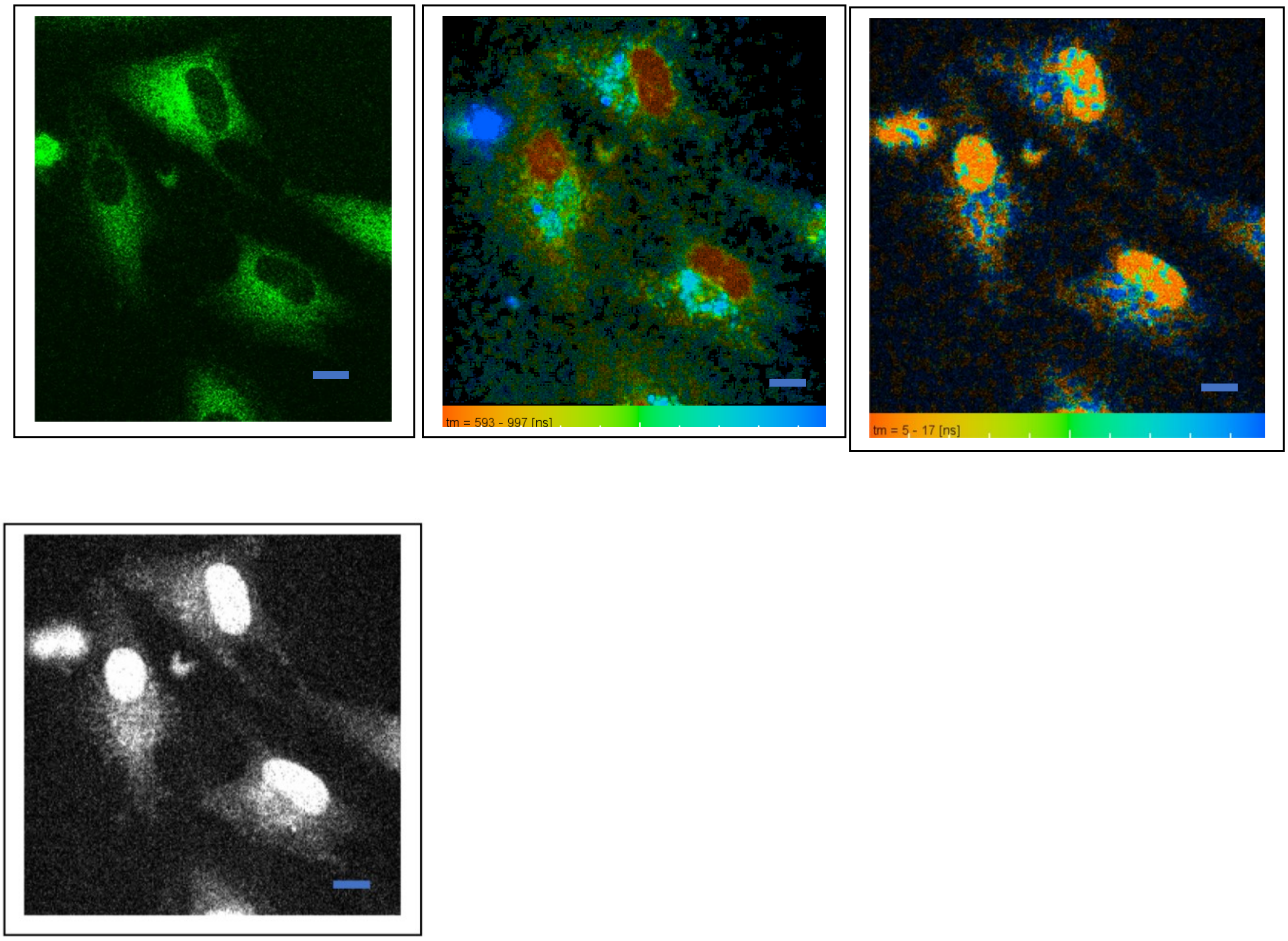

Figure S18: PLIM imaging of live HeLa cells incubated for 2 hr with 60 μM of **2** for 24hr followed by Hoechst and ER tracker co-staining, (100 x oil immersion microscope objective, NA 1.49) at room temperature. (a) confocal (b) PLIM channel (λ_{ex} = 405 nm/ $\lambda_{detection}$ = 600 nm long pass), (c, d) PLIM channel (λ_{ex} = 405 nm/ $\lambda_{detection}$ = 485 nm short pass). Scale bar 15 μm.

S3 References

1. Greetham, G. M.; Donaldson, P. M.; Nation, C. Sazanovich, S. V.; Clark, I. P.; Shaw, D. J.; Parker, A. W.; Towrie, M. *Appl. Spectr.,* **2016**, *70*, 645.
